# Supplementary material for: Peripheral leukocyte transcriptomic changes in preweaned Holstein heifer calves with varying stages of Bovine Respiratory Disease
Source: PLoS One. 2026 May 14;21(5):e0349348. doi: 10.1371/journal.pone.0349348 (PMC13175367; doi:10.1371/journal.pone.0349348)
Supplement: S1 Table — (DOCX) [file pone.0349348.s001.docx]

**S1 Table. Demographic characteristics of Holstein heifer calves selected for BRD comparisons.**

| *Healthy* ^a^ *vs Onset* ^b^ | | |  |  |  |
| --- | --- | --- | --- | --- | --- |
| Farm | **Year** | **Disease State** | **Week of Age** | **Sample Date** | **Calf ID** |
| A | 2021 | Healthy | 5 | 6/14/2021 | 92141 |
| A | 2021 | Healthy | 5 | 6/14/2021 | 92142 |
| A | 2021 | Healthy | 5 | 6/14/2021 | 92146 |
| A | 2021 | Healthy | 5 | 6/14/2021 | 92150 |
| A | 2021 | Healthy | 7 | 6/28/2021 | 92154 |
| A | 2022 | Healthy | 5 | 6/13/2022 | 96521 |
| A | 2022 | Healthy | 5 | 6/13/2022 | 96522 |
| A | 2022 | Healthy | 5 | 6/13/2022 | 96525 |
| A | 2022 | Healthy | 5 | 6/13/2022 | 96531 |
| A | 2022 | Healthy | 7 | 6/27/2022 | 96523 |
| A | 2022 | Healthy | 7 | 6/27/2022 | 96533 |
| A | 2022 | Healthy | 7 | 6/27/2022 | 96539 |
| A | 2022 | Healthy | 9 | 7/11/2022 | 96509 |
| B | 2021 | Healthy | 5 | 6/21/2021 | 3383 |
| B | 2021 | Healthy | 7 | 7/6/2021 | 3357 |
| B | 2021 | Healthy | 7 | 7/6/2021 | 3380 |
| B | 2022 | Healthy | 5 | 6/21/2022 | 4838 |
| B | 2022 | Healthy | 5 | 6/21/2022 | 4851 |
| B | 2022 | Healthy | 7 | 7/5/2022 | 4842 |
| B | 2022 | Healthy | 9 | 7/18/2022 | 4826 |
| B | 2022 | Healthy | 9 | 7/18/2022 | 4828 |
| B | 2022 | Healthy | 9 | 7/18/2022 | 4839 |
| A | 2022 | Onset | 5 | 6/13/2022 | 96519 |
| A | 2022 | Onset | 5 | 6/13/2022 | 96527 |
| A | 2021 | Onset | 7 | 6/28/2021 | 92155 |
| A | 2022 | Onset | 7 | 6/27/2022 | 96516 |
| A | 2022 | Onset | 7 | 6/27/2022 | 96520 |
| B | 2021 | Onset | 5 | 6/21/2021 | 3364 |
| B | 2021 | Onset | 5 | 6/21/2021 | 3369 |
| B | 2021 | Onset | 5 | 6/21/2021 | 3370 |
| B | 2021 | Onset | 5 | 6/21/2021 | 3373 |
| B | 2021 | Onset | 5 | 6/21/2021 | 3374 |
| B | 2021 | Onset | 5 | 6/21/2021 | 3376 |
| B | 2021 | Onset | 5 | 6/21/2021 | 3381 |
| B | 2022 | Onset | 5 | 6/21/2022 | 4832 |
| B | 2022 | Onset | 5 | 6/21/2022 | 4840 |
| B | 2021 | Onset | 7 | 7/6/2021 | 3360 |
| B | 2021 | Onset | 7 | 7/6/2021 | 3361 |
| B | 2021 | Onset | 7 | 7/6/2021 | 3363 |
| B | 2021 | Onset | 7 | 7/6/2021 | 3366 |
| B | 2021 | Onset | 7 | 7/6/2021 | 3367 |
| B | 2021 | Onset | 7 | 7/6/2021 | 3372 |
| B | 2021 | Onset | 7 | 7/6/2021 | 3375 |
| B | 2021 | Onset | 7 | 7/6/2021 | 3377 |
| B | 2022 | Onset | 7 | 7/5/2022 | 4822 |
| A | 2022 | Onset | 9 | 7/11/2022 | 96511 |
| B | 2022 | Onset | 5 | 6/21/2022 | 4833 |
| B | 2022 | Onset | 5 | 6/21/2022 | 4847 |
| B | 2022 | Onset | 7 | 7/5/2022 | 4841 |
| B | 2022 | Onset | 7 | 7/5/2022 | 4849 |
| B | 2022 | Onset | 9 | 7/18/2022 | 4830 |
| B | 2022 | Onset | 9 | 7/18/2022 | 4843 |
| B | 2022 | Onset | 9 | 7/18/2022 | 4850 |
|  |  |  |  |  |  |
| *Healthy vs Chronic* ^c^ | | |  |  |  |
| A | 2021 | Healthy | 7 | 6/28/2021 | 92141 |
| A | 2021 | Healthy | 7 | 6/28/2021 | 92142 |
| A | 2021 | Healthy | 7 | 6/28/2021 | 92146 |
| A | 2021 | Healthy | 7 | 6/28/2021 | 92150 |
| A | 2021 | Healthy | 9 | 7/12/2021 | 92154 |
| A | 2022 | Healthy | 9 | 7/11/2022 | 96509 |
| A | 2022 | Healthy | 5 | 6/13/2022 | 96521 |
| A | 2022 | Healthy | 5 | 6/13/2022 | 96522 |
| A | 2022 | Healthy | 7 | 6/27/2022 | 96523 |
| A | 2022 | Healthy | 5 | 6/13/2022 | 96525 |
| A | 2022 | Healthy | 5 | 6/13/2022 | 96531 |
| A | 2022 | Healthy | 7 | 6/27/2022 | 96533 |
| A | 2022 | Healthy | 7 | 6/27/2022 | 96539 |
| B | 2021 | Healthy | 9 | 7/19/2021 | 3357 |
| B | 2021 | Healthy | 9 | 7/19/2021 | 3380 |
| B | 2021 | Healthy | 7 | 7/6/2021 | 3383 |
| B | 2022 | Healthy | 9 | 7/18/2022 | 4826 |
| B | 2022 | Healthy | 9 | 7/18/2022 | 4828 |
| B | 2022 | Healthy | 5 | 6/21/2022 | 4838 |
| B | 2022 | Healthy | 9 | 7/18/2022 | 4839 |
| B | 2022 | Healthy | 7 | 7/5/2022 | 4842 |
| B | 2022 | Healthy | 5 | 6/21/2022 | 4851 |
| A | 2022 | Chronic | 7 | 6/27/2022 | 96513 |
| A | 2022 | Chronic | 9 | 7/11/2022 | 96514 |
| A | 2022 | Chronic | 9 | 7/11/2022 | 96515 |
| A | 2022 | Chronic | 7 | 6/27/2022 | 96528 |
| B | 2021 | Chronic | 9 | 7/19/2021 | 3372 |
| B | 2022 | Chronic | 7 | 7/5/2022 | 4824 |
| B | 2022 | Chronic | 7 | 7/5/2022 | 4835 |
| B | 2022 | Chronic | 9 | 7/18/2022 | 4845 |
| B | 2021 | Chronic | 9 | 7/19/2021 | 3360 |
| B | 2021 | Chronic | 9 | 7/19/2021 | 3361 |
| B | 2021 | Chronic | 9 | 7/19/2021 | 3363 |
| B | 2021 | Chronic | 7 | 7/6/2021 | 3364 |
| B | 2021 | Chronic | 9 | 7/19/2021 | 3366 |
| B | 2021 | Chronic | 7 | 7/6/2021 | 3374 |
| B | 2021 | Chronic | 7 | 7/6/2021 | 3381 |
| B | 2022 | Chronic | 7 | 7/5/2022 | 4836 |
| B | 2022 | Chronic | 9 | 7/18/2022 | 4840 |
|  |  |  |  |  |  |
| *Healthy vs Resolved* ^d^ | | |  |  |  |
| A | 2021 | Healthy | 7 | 6/28/2021 | 92141 |
| A | 2021 | Healthy | 7 | 6/28/2021 | 92142 |
| A | 2021 | Healthy | 7 | 6/28/2021 | 92146 |
| A | 2021 | Healthy | 7 | 6/28/2021 | 92150 |
| A | 2021 | Healthy | 9 | 7/12/2021 | 92154 |
| A | 2022 | Healthy | 9 | 7/11/2022 | 96509 |
| A | 2022 | Healthy | 5 | 6/13/2022 | 96521 |
| A | 2022 | Healthy | 5 | 6/13/2022 | 96522 |
| A | 2022 | Healthy | 7 | 6/27/2022 | 96523 |
| A | 2022 | Healthy | 5 | 6/13/2022 | 96525 |
| A | 2022 | Healthy | 5 | 6/13/2022 | 96531 |
| A | 2022 | Healthy | 7 | 6/27/2022 | 96533 |
| A | 2022 | Healthy | 7 | 6/27/2022 | 96539 |
| B | 2021 | Healthy | 9 | 7/19/2021 | 3357 |
| B | 2021 | Healthy | 9 | 7/19/2021 | 3380 |
| B | 2021 | Healthy | 7 | 7/6/2021 | 3383 |
| B | 2022 | Healthy | 9 | 7/18/2022 | 4826 |
| B | 2022 | Healthy | 9 | 7/18/2022 | 4828 |
| B | 2022 | Healthy | 5 | 6/21/2022 | 4838 |
| B | 2022 | Healthy | 9 | 7/18/2022 | 4839 |
| B | 2022 | Healthy | 7 | 7/5/2022 | 4842 |
| B | 2022 | Healthy | 5 | 6/21/2022 | 4851 |
| A | 2021 | Resolved | 9 | 7/12/2021 | 92155 |
| A | 2022 | Resolved | 9 | 7/11/2022 | 96516 |
| A | 2022 | Resolved | 7 | 6/27/2022 | 96519 |
| A | 2022 | Resolved | 9 | 7/11/2022 | 96520 |
| A | 2022 | Resolved | 7 | 6/27/2022 | 96527 |
| B | 2021 | Resolved | 9 | 7/19/2021 | 3367 |
| B | 2021 | Resolved | 7 | 7/6/2021 | 3369 |
| B | 2021 | Resolved | 7 | 7/6/2021 | 3370 |
| B | 2021 | Resolved | 7 | 7/6/2021 | 3373 |
| B | 2021 | Resolved | 9 | 7/19/2021 | 3375 |
| B | 2021 | Resolved | 9 | 7/19/2021 | 3377 |
| B | 2022 | Resolved | 9 | 7/18/2022 | 4822 |
| B | 2022 | Resolved | 7 | 7/5/2022 | 4827 |
| B | 2022 | Resolved | 9 | 7/18/2022 | 4829 |
| B | 2022 | Resolved | 7 | 7/5/2022 | 4832 |
| B | 2022 | Resolved | 9 | 7/18/2022 | 4834 |
| B | 2022 | Resolved | 9 | 7/18/2022 | 4836 |
| B | 2022 | Resolved | 7 | 7/5/2022 | 4844 |

^a^ Healthy defined as no evidence of lung pathology diagnosed via thoracic ultrasonography, no evidence of clinical respiratory signs (e.g., coughing, sneezing, nasal discharge), no evidence of scours, rectal temperature < 103.0°F (39.4°C), and no other reported health conditions by farm personnel.

^b^ Onset defined as initial identification of lobar consolidation diagnosed via thoracic ultrasonography.

^c^ Chronic defined as two or more weeks of lobar consolidation diagnosed via thoracic ultrasonography.

^d^ Resolved defined as no evidence of lobar pathology diagnosed via thoracic ultrasonography, no evidence of clinical respiratory signs, rectal temperature < 103.0°F (39.4°C), and no other reported health conditions by farm personnel.
