# Supplementary material for: Peripheral leukocyte transcriptomic changes in preweaned Holstein heifer calves with varying stages of Bovine Respiratory Disease
Source: PLoS One. 2026 May 14;21(5):e0349348. doi: 10.1371/journal.pone.0349348 (PMC13175367; doi:10.1371/journal.pone.0349348)
Supplement: S2 Table — (DOCX) [file pone.0349348.s002.docx]

**S2 Table. Summary of RNA integrity and purity parameters for submitted peripheral leukocyte transcriptomic samples.**

| Sample Name | Concentration (ng/ul) | RIN¹ |
| --- | --- | --- |
| MM | 15.36 | 2.3 |
| WW | 11.75 | 3.3 |
| BBB | 37.13 | 3.3 |
| GG512 | 36.54 | 3.3 |
| XX | 14.7 | 3.5 |
| KK | 22.46 | 3.6 |
| RR | 48.9 | 3.8 |
| LL | 32.87 | 3.9 |
| U | 52.39 | 4.1 |
| JJ | 43.67 | 4.1 |
| QQ | 39.67 | 4.2 |
| NN | 28.42 | 4.5 |
| AAA | 33.42 | 4.6 |
| E | 22.14 | 4.9 |
| TT | 20.89 | 4.9 |
| Q471 | 23.31 | 4.9 |
| W | 21.9 | 5 |
| II | 24.13 | 5 |
| UU | 38.6 | 5.1 |
| ZZ | 41.73 | 5.1 |
| D | 21.6 | 5.2 |
| F | 25.1 | 5.2 |
| SS | 20.81 | 5.3 |
| AA | 50.49 | 5.4 |
| B | 45.86 | 5.6 |
| PP | 47.51 | 5.6 |
| P | 41.03 | 5.8 |
| BB504 | 123.14 | 5.8 |
| SS543 | 57.56 | 5.8 |
| R | 28.56 | 6 |
| DD | 60.83 | 6 |
| VV | 42.88 | 6 |
| O466 | 81.57 | 6 |
| P470 | 76.21 | 6 |
| FFF583 | 107.14 | 6 |
| HH | 60.22 | 6.1 |
| TT544 | 67.74 | 6.1 |
| ZZ569 | 63.55 | 6.1 |
| CCC578 | 90.74 | 6.1 |
| C | 24.7 | 6.3 |
| Q | 44.58 | 6.3 |
| T | 32.88 | 6.3 |
| OO | 22.66 | 6.3 |
| FF510 | 79.85 | 6.3 |
| XX564 | 79.28 | 6.3 |
| S | 20.97 | 6.4 |
| GG | 26.85 | 6.4 |
| U484 | 93.62 | 6.4 |
| YY566 | 84.49 | 6.4 |
| I452 | 24.05 | 6.5 |
| AA503 | 135.38 | 6.5 |
| V | 26.09 | 6.6 |
| CC506 | 67.84 | 6.6 |
| NN520 | 73.11 | 6.6 |
| VV560 | 135.38 | 6.6 |
| G | 28.51 | 6.7 |
| H447 | 79.07 | 6.7 |
| Z | 33.42 | 6.8 |
| BB | 64.98 | 6.8 |
| S479 | 69.58 | 6.8 |
| DDD580 | 109.76 | 6.8 |
| FF | 28.73 | 6.9 |
| T483 | 66.47 | 6.9 |
| Z500 | 83.47 | 6.9 |
| N | 40.61 | 7 |
| RR526 | 141.68 | 7 |
| MM519 | 35.45 | 7.1 |
| WW563 | 121.55 | 7.1 |
| AAA570 | 36.47 | 7.1 |
| CC | 30.5 | 7.2 |
| EE | 27.52 | 7.2 |
| R475 | 54.07 | 7.3 |
| DD507 | 66.85 | 7.3 |
| JJ516 | 22.24 | 7.3 |
| EE509 | 48.09 | 7.4 |
| BBB575 | 82.97 | 7.4 |
| I | 40.05 | 7.5 |
| Y | 30.15 | 7.5 |
| J453 | 53.02 | 7.5 |
| KK517 | 57 | 7.5 |
| N464 | 36.87 | 7.6 |
| M | 87.33 | 7.8 |
| II515 | 36 | 7.8 |
| PP523 | 115.23 | 7.8 |
| K456 | 77.26 | 7.9 |
| QQ524 | 64.5 | 7.9 |
| H | 34.12 | 8 |
| HH513 | 25.64 | 8 |
| LL518 | 30.78 | 8.1 |
| J | 41.29 | 8.3 |
| K | 126.49 | 8.4 |
| L457 | 102.37 | 8.4 |
| B411 | 43.35 | 8.5 |
| A410 | 59.76 | 8.6 |
| D419 | 131.6 | 8.8 |
| C415 | 33.4 | 9 |

^1^RNA Integrity Number (RIN) is a standardized score that indicates the integrity of RNA samples.
