# Supplementary material for: Peripheral leukocyte transcriptomic changes in preweaned Holstein heifer calves with varying stages of Bovine Respiratory Disease
Source: PLoS One. 2026 May 14;21(5):e0349348. doi: 10.1371/journal.pone.0349348 (PMC13175367; doi:10.1371/journal.pone.0349348)
Supplement: S3 Table — (DOCX) [file pone.0349348.s003.docx]

**S3 Table. Descriptive summary table of differentially expressed genes (DEGs: n = 163; FDR < 0.05 and |logFC| > 1) identified for *Healthy* vs *Onset*.**

| Gene Symbol | Description | logFC | logCPM | *p*-value | FDR |
| --- | --- | --- | --- | --- | --- |
| LOC112444532 | Interleukin-3 receptor subunit alpha | 2.52 | 3.55 | 2.71E-08 | 0.001 |
| SHROOM4 | Protein Shroom4 isoform X2 | 1.43 | 4.01 | 1.21E-07 | 0.002 |
| IL3RA | Interleukin-3 receptor subunit alpha isoform X2 | 2.44 | 3.05 | 1.34E-07 | 0.002 |
| LOC112444489 | Uncharacterized protein LOC112444489 | 1.89 | 0.18 | 2.37E-07 | 0.002 |
| IL3RA | Interleukin-3 receptor subunit alpha isoform X1 | 2.24 | 5.25 | 3.14E-07 | 0.003 |
| RAB3IP | Predicted: RAB3A interacting protein isoform X7 | 1.78 | 3.95 | 3.78E-07 | 0.003 |
| CFB | Complement factor B precursor | 2.11 | 3.40 | 5.22E-07 | 0.003 |
| IL1RAP | Interleukin-1 receptor accessory protein precursor | 1.19 | 3.30 | 1.61E-06 | 0.003 |
| IL2RA | Interleukin-2 receptor subunit alpha isoform X2 | 1.23 | 6.56 | 1.02E-06 | 0.003 |
| LOC112447850 | Uncharacterized protein LOC112447850 | 1.34 | 3.60 | 1.65E-06 | 0.003 |
| FOXRED1 | FAD-dependent oxidoreductase domain-containing protein 1 | 1.44 | 6.75 | 1.42E-06 | 0.003 |
| SLC28A3 | Solute carrier family 28 member 3 isoform X1 | 1.48 | 6.47 | 1.73E-06 | 0.003 |
| KBTBD7 | Kelch repeat and BTB domain-containing protein 7 | 1.49 | 5.22 | 1.46E-06 | 0.003 |
| LIMK2 | LIM domain kinase 2 isoform X2 | 1.51 | 5.69 | 1.04E-06 | 0.003 |
| LCN2 | Neutrophil gelatinase-associated lipocalin | 1.75 | 3.55 | 1.74E-06 | 0.003 |
| LCN2 | Neutrophil gelatinase-associated lipocalin | 1.82 | 5.14 | 1.68E-06 | 0.003 |
| ACTN1 | Alpha-actinin-1 isoform X3 | 2.07 | 4.89 | 8.88E-07 | 0.003 |
| RAB3IP | Rab-3A-interacting protein isoform X1 | 2.09 | 3.14 | 1.52E-06 | 0.003 |
| ALPL | Alkaline phosphatase, tissue-nonspecific isozyme precursor | 2.11 | 6.73 | 1.67E-06 | 0.003 |
| LOC100847256 | Uncharacterized protein LOC100847256 isoform X1 | 2.61 | 4.21 | 1.24E-06 | 0.003 |
| SLCO4C1 | Solute carrier organic anion transporter family member 4C1 isoform X1 | 1.05 | 4.30 | 2.32E-06 | 0.004 |
| WIPI1 | WD repeat domain phosphoinositide-interacting protein 1 | 1.11 | 3.78 | 2.35E-06 | 0.004 |
| MEGF9 | Multiple epidermal growth factor-like domains protein 9 | 1.44 | 7.29 | 2.22E-06 | 0.004 |
| LIMK2 | LIM domain kinase 2 isoform X5 | 1.45 | 1.33 | 2.27E-06 | 0.004 |
| RAB3IP | Predicted: RAB3A interacting protein isoform X8 | 1.75 | 4.02 | 2.41E-06 | 0.004 |
| KCBD1 | Potassium voltage-gated channel subfamily D member 1 isoform X2 | -2.75 | 1.36 | 2.66E-06 | 0.004 |
| DNMT3L | DNA (cytosine-5)-methyltransferase 3-like | 1.38 | 0.20 | 3.04E-06 | 0.004 |
| CDKN2B | Cyclin-dependent kinase 4 inhibitor B | 1.34 | 2.12 | 3.65E-06 | 0.005 |
| FAM107B | Protein FAM107B isoform X2 | 1.62 | 5.83 | 4.03E-06 | 0.005 |
| TMPRSS3 | Transmembrane protease serine 3 | 1.99 | 0.27 | 3.98E-06 | 0.005 |
| S100A12 | Protein S100-A12 isoform X1 | 1.66 | 11.34 | 4.39E-06 | 0.005 |
| IL1R2 | Interleukin-1 receptor type 2 precursor | 2.07 | 3.73 | 4.42E-06 | 0.005 |
| VDR | Vitamin D3 receptor isoform X1 | 1.04 | 3.31 | 5.00E-06 | 0.006 |
| DYSF | Dysferlin | 1.12 | 8.04 | 5.10E-06 | 0.006 |
| ITGB5 | Integrin beta-5 precursor | -1.22 | 1.99 | 6.26E-06 | 0.006 |
| GPR27 | Probable G-protein coupled receptor 27 | 1.08 | 3.95 | 6.29E-06 | 0.006 |
| ALOX5AP | Arachidonate 5-lipoxygenase-activating protein | 1.18 | 7.22 | 6.00E-06 | 0.006 |
| S100A9 | Protein S100-A9 isoform X1 | 1.51 | 8.86 | 5.97E-06 | 0.006 |
| LIMK2 | LIM domain kinase 2 | 1.57 | 3.58 | 5.88E-06 | 0.006 |
| LOC505972 | Fibroin heavy chain isoform X3 | 2.23 | 3.50 | 5.72E-06 | 0.006 |
| ALPL | Alkaline phosphatase, tissue-nonspecific isozyme isoform X1 | 2.24 | 3.80 | 6.10E-06 | 0.006 |
| LOC508459 | Adhesion G protein-coupled receptor E1 isoform X14 | 1.57 | 6.64 | 6.70E-06 | 0.006 |
| LOC505972 | Fibroin heavy chain isoform X1 | 2.08 | 3.48 | 6.59E-06 | 0.006 |
| ADGRG3 | Adhesion G protein-coupled receptor G3 isoform X2 | 1.14 | 5.67 | 7.36E-06 | 0.006 |
| CD24 | Signal transducer CD24 | 1.26 | 10.09 | 7.61E-06 | 0.006 |
| S100A9 | Protein S100-A9 isoform X1 | 1.42 | 11.03 | 7.54E-06 | 0.006 |
| CPNE2 | Copine-2 isoform X2 | 1.90 | 2.91 | 7.55E-06 | 0.006 |
| PTPN5 | Tyrosine-protein phosphatase non-receptor type 5 isoform X2 | 1.35 | 5.17 | 8.13E-06 | 0.006 |
| ALPL | Alkaline phosphatase, tissue-nonspecific isozyme isoform X1 | 2.11 | 2.57 | 8.16E-06 | 0.006 |
| S100A8 | Protein S100-A8 | 1.60 | 9.49 | 8.87E-06 | 0.006 |
| WNK1 | Serine/threonine-protein kinase WNK1 isoform X16 | -4.09 | -0.84 | 9.56E-06 | 0.007 |
| TMEM229B | Transmembrane protein 229B isoform X1 | 1.33 | 4.11 | 1.03E-05 | 0.007 |
| KREMEN1 | Kremen protein 1 isoform X2 | 1.48 | 6.10 | 1.02E-05 | 0.007 |
| IL1R2 | Interleukin-1 receptor type 2 isoform X1 | 2.05 | 3.67 | 1.04E-05 | 0.007 |
| RYR1 | Ryanodine receptor 1 isoform X1 | 2.03 | 3.92 | 1.08E-05 | 0.007 |
| STX11 | Syntaxin-11 isoform X1 | 1.60 | 2.61 | 1.12E-05 | 0.007 |
| LPCAT2 | Lysophosphatidylcholine acyltransferase 2 isoform X1 | 1.07 | 4.82 | 1.28E-05 | 0.008 |
| ADGRG3 | Adhesion G protein-coupled receptor G3 isoform X1 | 1.25 | 4.69 | 1.42E-05 | 0.008 |
| EIF4E3 | Eukaryotic translation initiation factor 4E type 3 | 1.06 | 5.83 | 1.59E-05 | 0.009 |
| RFC1 | Replication factor C subunit 1 isoform X1 | -2.14 | -1.28 | 1.90E-05 | 0.010 |
| LOC112441484 | Interferon-induced transmembrane protein 3-like | 1.29 | 7.62 | 1.88E-05 | 0.010 |
| LOC107132719 | Uncharacterized protein LOC107132719 isoform X1 | 1.65 | 0.22 | 1.88E-05 | 0.010 |
| HIST1H1C | Histone H1.2 | 1.08 | 7.93 | 1.94E-05 | 0.010 |
| LIMK2 | LIM domain kinase 2 isoform X2 | 1.50 | 3.02 | 2.26E-05 | 0.011 |
| PRDX5 | Peroxiredoxin-5, mitochondrial precursor | 1.04 | 7.25 | 2.52E-05 | 0.011 |
| LOC112442060 | Uncharacterized protein LOC112442060 | 1.10 | 2.27 | 2.48E-05 | 0.011 |
| LIMK2 | LIM domain kinase 2 isoform X4 | 1.46 | 5.07 | 2.56E-05 | 0.011 |
| LOC508459 | Adhesion G protein-coupled receptor E1 isoform X16 | 1.70 | 4.16 | 2.33E-05 | 0.011 |
| TIAM2 | T-lymphoma invasion and metastasis-inducing protein 2 isoform X3 | 1.81 | 2.78 | 2.47E-05 | 0.011 |
| LOC783362 | Uncharacterized protein LOC783362 isoform X1 | 1.98 | 1.27 | 2.57E-05 | 0.011 |
| LOC783362 | Uncharacterized protein LOC783362 isoform X2 | 1.98 | 1.27 | 2.57E-05 | 0.011 |
| RAB3IP | Rab-3A-interacting protein isoform X1 | 2.69 | 0.09 | 2.45E-05 | 0.011 |
| CELSR3 | Cadherin EGF LAG seven-pass G-type receptor 3 precursor | 1.05 | 0.24 | 2.76E-05 | 0.012 |
| CCDC191 | Coiled-coil domain-containing protein 191 isoform X1 | 1.52 | 3.71 | 2.82E-05 | 0.012 |
| LOC112449349 | Uncharacterized protein LOC112449349 isoform X2 | 1.10 | 4.47 | 2.91E-05 | 0.012 |
| IL18BP | Interleukin-18-binding protein | 1.17 | 4.18 | 3.14E-05 | 0.012 |
| ACTN1 | Alpha-actinin-1 isoform X4 | 1.22 | 3.05 | 3.35E-05 | 0.012 |
| TCN1 | Transcobalamin-1 precursor | 1.25 | 8.75 | 3.12E-05 | 0.012 |
| CA4 | Carbonic anhydrase 4 precursor | 1.54 | 3.16 | 3.34E-05 | 0.012 |
| BMX | Cytoplasmic tyrosine-protein kinase BMX isoform X2 | 1.87 | 2.96 | 3.36E-05 | 0.012 |
| MYRFL | Myelin regulatory factor-like protein isoform X1 | 2.09 | 0.62 | 3.29E-05 | 0.012 |
| SYNE1 | Nesprin-1 isoform X11 | 3.22 | 2.44 | 3.31E-05 | 0.012 |
| STX11 | Syntaxin-11 isoform X1 | 1.41 | 3.08 | 3.51E-05 | 0.012 |
| CNST | Consortin isoform X1 | -3.72 | -1.24 | 3.58E-05 | 0.012 |
| LOC101907697 | Predicted: Homeobox protein CDX-1-like isoform X3 | 1.29 | 0.13 | 3.66E-05 | 0.013 |
| CASP8AP2 | CASP8-associated protein 2 isoform X1 | 1.79 | -0.34 | 3.81E-05 | 0.013 |
| OSCAR | Osteoclast-associated immunoglobulin-like receptor isoform X1 | 1.07 | 7.16 | 3.85E-05 | 0.013 |
| SOCS7 | Suppressor of cytokine signaling 7 isoform X1 | 6.57 | -0.12 | 4.17E-05 | 0.014 |
| OLFM4 | Olfactomedin-4 | 1.17 | 4.03 | 4.57E-05 | 0.015 |
| PROK2 | Prokineticin-2 isoform X1 | 1.17 | 4.22 | 4.79E-05 | 0.015 |
| MMP9 | Matrix metalloproteinase-9 precursor | 1.13 | 5.73 | 4.84E-05 | 0.015 |
| TRPC5 | Short transient receptor potential channel 5 | 1.09 | 1.79 | 4.95E-05 | 0.015 |
| HEATR5A | HEAT repeat-containing protein 5A isoform X1 | 2.55 | 0.90 | 4.97E-05 | 0.015 |
| CCL16 | C-C motif chemokine 16 | 1.10 | 7.28 | 5.02E-05 | 0.015 |
| BMX | Cytoplasmic tyrosine-protein kinase BMX | 1.41 | 4.23 | 5.21E-05 | 0.015 |
| LOC529196 | C-C chemokine receptor type 1-like isoform X1 | 1.48 | 1.96 | 5.85E-05 | 0.016 |
| LOC786348 | Serpin B4-like | 2.86 | 3.17 | 5.93E-05 | 0.016 |
| LOC112441663 | Uncharacterized protein LOC112441663 isoform X1 | 1.43 | 4.58 | 6.13E-05 | 0.016 |
| DHRSX | Dehydrogenase/reductase SDR family member on chromosome X | 1.07 | 2.62 | 6.25E-05 | 0.016 |
| DEFB4A | Beta-defensin 4 precursor | 1.67 | 0.41 | 6.36E-05 | 0.017 |
| CPNE2 | Copine-2 | 1.54 | 2.29 | 6.47E-05 | 0.017 |
| SYNJ1 | Synaptojanin-1 isoform X8 | 5.54 | -0.38 | 6.51E-05 | 0.017 |
| BMX | Cytoplasmic tyrosine-protein kinase BMX isoform X1 | 1.30 | 4.13 | 6.92E-05 | 0.018 |
| IL21R | Interleukin-21 receptor isoform X1 | 2.39 | 4.28 | 7.46E-05 | 0.019 |
| NOL3 | Nucleolar protein 3 | 1.36 | 1.03 | 7.58E-05 | 0.019 |
| IL18R1 | Interleukin-18 receptor 1 isoform X1 | 1.60 | 0.51 | 8.29E-05 | 0.020 |
| KIAA1522 | Uncharacterized protein KIAA1522 homolog isoform X2 | -2.81 | -1.01 | 9.02E-05 | 0.021 |
| SLC25A37 | Mitoferrin-1 isoform X1 | 1.04 | 5.67 | 8.82E-05 | 0.021 |
| RGS3 | Regulator of G-protein signaling 3 isoform X1 | 1.62 | -0.91 | 9.04E-05 | 0.021 |
| MOCS2 | Molybdopterin synthase catalytic subunit isoform X1 | 1.18 | 0.07 | 9.12E-05 | 0.021 |
| SLCO4A1 | Solute carrier organic anion transporter family member 4A1 isoform X2 | 1.24 | 4.60 | 9.58E-05 | 0.022 |
| LOC100848368 | Uncharacterized protein LOC100848368 isoform X2 | 1.43 | 2.47 | 9.61E-05 | 0.022 |
| RNF150 | RING finger protein 150 isoform X1 | -3.52 | -0.25 | 9.67E-05 | 0.022 |
| THY1 | Thy-1 membrane glycoprotein precursor | 1.77 | 2.61 | 9.84E-05 | 0.022 |
| ABCA7 | Predicted: ATP binding cassette subfamily A member 7 | 1.15 | 0.73 | 1.09E-04 | 0.024 |
| SLC6A9 | Sodium- and chloride-dependent glycine transporter 1 isoform 1 | 2.14 | 2.24 | 1.09E-04 | 0.024 |
| SLC45A4 | Solute carrier family 45 member 4 isoform X2 | 1.06 | 3.12 | 1.15E-04 | 0.024 |
| USP48 | Ubiquitin carboxyl-terminal hydrolase 48 isoform X5 | -2.39 | -1.02 | 1.29E-04 | 0.027 |
| NADSYN1 | Glutamine-dependent NAD(+) synthetase isoform X7 | 1.37 | 2.49 | 1.30E-04 | 0.027 |
| HACE1 | E3 ubiquitin-protein ligase HACE1 isoform X5 | 5.07 | -1.76 | 1.32E-04 | 0.027 |
| TGIF2 | Homeobox protein TGIF2 isoform X1 | -1.17 | 1.63 | 1.39E-04 | 0.028 |
| LOC100336589 | Killer cell immunoglobulin-like receptor 2DL5A isoform X4 | 1.28 | 5.08 | 1.40E-04 | 0.028 |
| SLC28A3 | Solute carrier family 28 member 3 isoform X1 | 1.26 | 5.62 | 1.42E-04 | 0.028 |
| CPNE2 | Copine-2 isoform X1 | 1.44 | 3.70 | 1.53E-04 | 0.029 |
| UBAP2L | Ubiquitin-associated protein 2-like isoform X20 | 2.23 | 2.02 | 1.55E-04 | 0.029 |
| S100A12 | Protein S100-A12 | 1.06 | 8.09 | 1.58E-04 | 0.030 |
| DNM2 | Dynamin-2 isoform X9 | 1.48 | 1.45 | 1.59E-04 | 0.030 |
| GLT1D1 | Glycosyltransferase 1 domain-containing protein 1 isoform X1 | 1.07 | 3.87 | 1.61E-04 | 0.030 |
| TNFAIP6 | Tumor necrosis factor-inducible gene 6 protein isoform X1 | 1.38 | 1.85 | 1.76E-04 | 0.032 |
| ZFR | Zinc finger RNA-binding protein isoform X3 | -4.14 | 0.67 | 1.78E-04 | 0.032 |
| BCL6 | B-cell lymphoma 6 protein isoform X1 | 1.02 | 7.89 | 1.88E-04 | 0.033 |
| S100A9 | Protein S100-A9 isoform X1 | 1.28 | 8.40 | 1.92E-04 | 0.033 |
| UPP1 | Uridine phosphorylase 1 | 1.37 | 6.17 | 1.95E-04 | 0.034 |
| SLCO4C1 | Solute carrier organic anion transporter family member 4C1 | 1.09 | 3.12 | 1.97E-04 | 0.034 |
| LOC112447832 | Uncharacterized protein LOC112447832 isoform X2 | 1.74 | 2.43 | 2.05E-04 | 0.035 |
| SLC28A3 | Solute carrier family 28 member 3 | 1.83 | 3.58 | 2.05E-04 | 0.035 |
| LOC104974144 | Uncharacterized protein LOC104974144 isoform X1 | 2.16 | 1.94 | 2.06E-04 | 0.035 |
| PABPC4 | Polyadenylate-binding protein 4 isoform X6 | -2.06 | 0.91 | 2.13E-04 | 0.035 |
| LOC112445945 | Uncharacterized protein LOC112445945 isoform X2 | 1.10 | 0.79 | 2.30E-04 | 0.037 |
| TTC1 | Tetratricopeptide repeat protein 1 isoform X1 | 1.01 | 2.90 | 2.34E-04 | 0.037 |
| MS4A14 | Membrane-spanning 4-domains subfamily A member 14 | -1.01 | 0.28 | 2.38E-04 | 0.037 |
| IL1RAP | Interleukin-1 receptor accessory protein isoform X6 | 1.38 | 4.03 | 2.40E-04 | 0.037 |
| NBEA | Neurobeachin isoform X3 | -1.85 | 0.99 | 2.44E-04 | 0.037 |
| IL15RA | Interleukin-15 receptor subunit alpha isoform X2 | 1.27 | 3.25 | 2.51E-04 | 0.037 |
| RAB3IP | Rab-3A-interacting protein isoform X1 | 1.66 | 1.63 | 2.55E-04 | 0.038 |
| UBAP1 | Ubiquitin-associated protein 1 isoform X1 | 1.16 | 4.17 | 2.57E-04 | 0.038 |
| SGCA | Alpha-sarcoglycan isoform X1 | 3.39 | -1.56 | 2.74E-04 | 0.040 |
| LOC100847256 | Uncharacterized protein LOC100847256 isoform X2 | 1.75 | 3.17 | 2.75E-04 | 0.040 |
| AATK | Serine/threonine-protein kinase LMTK1 isoform X3 | 1.81 | 1.57 | 2.84E-04 | 0.041 |
| TIAM2 | T-lymphoma invasion and metastasis-inducing protein 2 isoform X1 | 1.04 | 3.53 | 2.93E-04 | 0.042 |
| C3H1orf162 | Transmembrane protein C1orf162 homolog isoform X1 | 1.04 | 2.65 | 2.98E-04 | 0.042 |
| ALPL | Alkaline phosphatase, tissue-nonspecific isozyme isoform X1 | 2.21 | 1.45 | 3.02E-04 | 0.042 |
| JARID2 | Protein Jumonji isoform X3 | 4.58 | 0.49 | 3.07E-04 | 0.043 |
| ZDHHC2 | Palmitoyltransferase ZDHHC2 | 1.12 | 4.74 | 3.13E-04 | 0.043 |
| C7H19orf38 | Protein HIDE1 isoform X6 | 1.15 | 1.22 | 3.23E-04 | 0.044 |
| CDC42BPA | Serine/threonine-protein kinase MRCK alpha isoform X1 | 4.04 | 0.44 | 3.24E-04 | 0.044 |
| LOC112449349 | Uncharacterized protein LOC112449349 isoform X3 | 1.22 | 2.21 | 3.48E-04 | 0.045 |
| SLC6A2 | Sodium-dependent noradrenaline transporter isoform X2 | 1.38 | 1.77 | 3.50E-04 | 0.045 |
| NFIL3 | Nuclear factor Interleukin-3-regulated protein isoform X1 | 1.03 | 5.92 | 3.69E-04 | 0.047 |
| MIA2 | Endoplasmic reticulum export factor CTAGE5 isoform X15 | -1.64 | -0.94 | 3.71E-04 | 0.047 |
| CDH23 | Cadherin-23 isoform X9 | -1.33 | 0.96 | 3.80E-04 | 0.048 |
| RAB3IP | Rab-3A-interacting protein | 2.01 | 4.18 | 3.84E-04 | 0.048 |
| LOC618664 | Ecto-ADP-ribosyltransferase 5 isoform X1 | 1.10 | 5.82 | 3.96E-04 | 0.048 |

LogFC, Log Fold Change; LogCPM, Log Counter Per Million; FDR, False Discovery Rate
