# Supplementary material for: Peripheral leukocyte transcriptomic changes in preweaned Holstein heifer calves with varying stages of Bovine Respiratory Disease
Source: PLoS One. 2026 May 14;21(5):e0349348. doi: 10.1371/journal.pone.0349348 (PMC13175367; doi:10.1371/journal.pone.0349348)
Supplement: S4 Table — (DOCX) [file pone.0349348.s004.docx]

**S4 Table. Descriptive summary table of differentially expressed genes (****DEGs: n = 27; FDR < 0.05 and |logFC| > 1) identified for *Healthy* vs *Chronic*.**

| Gene Symbol | Description | logFC | logCPM | *p-*value | FDR |
| --- | --- | --- | --- | --- | --- |
| SPTAN1 | Spectrin alpha chain, non-erythrocytic 1 isoform X1 | -3.86 | 0.62 | 3.87E-05 | 0.042 |
| CLGN | Calmegin precursor | -1.18 | 2.83 | 2.15E-05 | 0.033 |
| LOC100848368 | Uncharacterized protein LOC100848368 | 1.02 | 3.78 | 4.92E-05 | 0.047 |
| SESN2 | Sestrin-2 | 1.05 | 2.54 | 5.20E-05 | 0.047 |
| IL2RA | Interleukin-2 receptor subunit alpha isoform X2 | 1.05 | 6.28 | 3.35E-05 | 0.041 |
| LOC281376 | Plasminogen activator inhibitor 2 | 1.09 | 2.55 | 1.57E-05 | 0.027 |
| BMX | Cytoplasmic tyrosine-protein kinase BMX | 1.17 | 3.95 | 4.59E-05 | 0.045 |
| LDAF1 | Lipid droplet assembly factor 1 | 1.18 | 1.79 | 8.27E-06 | 0.021 |
| CCDC191 | Coiled-coil domain-containing protein 191 isoform X1 | 1.29 | 3.33 | 3.65E-05 | 0.042 |
| CDKN2B | Cyclin-dependent kinase 4 inhibitor B | 1.29 | 2.06 | 2.78E-05 | 0.038 |
| PREX1 | Phosphatidylinositol 3,4,5-trisphosphate-dependent Rac exchanger 1 protein isoform X2 | 1.33 | 5.24 | 1.97E-07 | 0.004 |
| IL3RA | Interleukin-3 receptor subunit alpha isoform X1 | 1.53 | 4.68 | 7.11E-06 | 0.020 |
| CA4 | Carbonic anhydrase 4 precursor | 1.60 | 3.16 | 1.58E-05 | 0.027 |
| LOC112444532 | Interleukin-3 receptor subunit alpha | 1.73 | 3.02 | 6.30E-06 | 0.020 |
| RYR1 | Ryanodine receptor 1 isoform X1 | 1.74 | 3.43 | 4.09E-05 | 0.042 |
| LOC104972045 | Uncharacterized protein LOC104972045 | 1.78 | 2.69 | 3.99E-05 | 0.042 |
| LOC112441663 | Uncharacterized protein LOC112441663 | 1.81 | 4.92 | 4.32E-05 | 0.043 |
| IL1R2 | Interleukin-1 receptor type 2 isoform X1 | 1.97 | 3.33 | 3.99E-07 | 0.004 |
| LOC104973468 | Uncharacterized protein LOC104973468 isoform X2 | 2.35 | 0.35 | 1.45E-05 | 0.027 |
| IFI27 | Interferon alpha-inducible protein 27 | 2.49 | 4.18 | 7.29E-06 | 0.020 |
| GPR158 | Predicted: G protein-coupled receptor 158 isoform X2 | 2.56 | 1.25 | 5.02E-06 | 0.020 |
| LOC100847171 | Uncharacterized protein LOC100847171 isoform X5 | 2.70 | 2.28 | 2.11E-05 | 0.033 |
| LOC783362 | Uncharacterized protein LOC783362 isoform X1 | 2.77 | 2.03 | 2.34E-06 | 0.013 |
| LOC783362 | Uncharacterized protein LOC783362 isoform X2 | 2.77 | 2.03 | 2.34E-06 | 0.013 |
| MITF | Microphthalmia-associated transcription factor isoform X9 | 2.92 | 2.36 | 5.18E-05 | 0.047 |
| LOC786942 | Uncharacterized protein LOC786942 | 3.22 | 0.21 | 5.69E-06 | 0.020 |
| PARD3 | Partitioning defective 3 homolog isoform X3 | 3.24 | 0.10 | 2.88E-05 | 0.038 |

LogFC, Log Fold Change; LogCPM, Log Counter Per Million; FDR, False Discovery Rate
