# Supplementary material for: Peripheral leukocyte transcriptomic changes in preweaned Holstein heifer calves with varying stages of Bovine Respiratory Disease
Source: PLoS One. 2026 May 14;21(5):e0349348. doi: 10.1371/journal.pone.0349348 (PMC13175367; doi:10.1371/journal.pone.0349348)
Supplement: S5 Table — (DOCX) [file pone.0349348.s005.docx]

**S5 Table. Descriptive summary table of random forest gene features (n = 51) identified for *Healthy* vs *Onset*.**

| Gene Symbol | Description | Importance Score |
| --- | --- | --- |
| CNTRL | Centriolin isoform X4 | 0.040 |
| LOC508459 | Adhesion G protein-coupled receptor E1 isoform X14 | 0.040 |
| MS4A15 | Membrane-spanning 4-domains subfamily A member 15 | 0.040 |
| ANKRD33B | Ankyrin repeat domain-containing protein 33B isoform X1 | 0.040 |
| FLOT2 | Flotillin-2 | 0.040 |
| LOC100847661 | Bos taurus uncharacterized | 0.033 |
| PPM1F | Protein phosphatase 1F isoform X2 | 0.033 |
| LOC100847256 | Bos taurus uncharacterized isoform X1 | 0.027 |
| LOC104975925 | Bos taurus uncharacterized isoform X1 | 0.027 |
| LOC112442364 | Bos taurus uncharacterized | 0.027 |
| LOC112441875 | Bos taurus uncharacterized isoform X2 | 0.027 |
| LOC508459 | Adhesion G protein-coupled receptor E1 isoform X16 | 0.027 |
| AP1G2 | AP-1 complex subunit gamma-like 2 isoform X5 | 0.027 |
| GGTA1 | N-acetyllactosaminide alpha-1,3-galactosyltransferase isoform X5 | 0.027 |
| PAX8 | Paired box protein Pax-8 isoform X1 | 0.027 |
| TNIP2 | TNFAIP3-interacting protein 2 isoform X1 | 0.027 |
| HPS1 | BLOC-3 complex member HPS1 | 0.027 |
| TTLL12 | Tubulin--tyrosine ligase-like protein 12 | 0.027 |
| LOC104973586 | Bos taurus uncharacterized | 0.027 |
| PTPRF | Receptor-type tyrosine-protein phosphatase F isoform X4 | 0.020 |
| PICALM | Phosphatidylinositol-binding clathrin assembly protein isoform X12 | 0.020 |
| FHOD1 | FH1/FH2 domain-containing protein 1 isoform X1 | 0.020 |
| RPL6 | 60S ribosomal protein L6 isoform X1 | 0.020 |
| ZNF10 | Zinc finger protein 10 isoform X3 | 0.020 |
| IL21R | Interleukin-21 receptor isoform X1 | 0.020 |
| TAMALIN | Protein TAMALIN | 0.020 |
| ASIP | Agouti-signaling protein precursor | 0.020 |
| HSPA2 | Heat shock-related 70 kDa protein 2 | 0.020 |
| GZMB | Duodenase-1 precursor | 0.020 |
| MSRA | Mitochondrial peptide methionine sulfoxide reductase | 0.020 |
| HNRNPC | Heterogeneous nuclear ribonucleoproteins C1/C2 isoform X1 | 0.013 |
| TARBP1 | Probable methyltransferase TARBP1 isoform X1 | 0.013 |
| RNF44 | RING finger protein 44 isoform X3 | 0.013 |
| ARL11 | ADP-ribosylation factor-like protein 11 | 0.013 |
| GATA2 | Endothelial transcription factor GATA-2 | 0.013 |
| KLHL7 | Kelch-like protein 7 | 0.013 |
| CDKN2B | Cyclin-dependent kinase 4 inhibitor B | 0.013 |
| PHOSPHO2 | Pyridoxal phosphate phosphatase PHOSPHO2 isoform X1 | 0.013 |
| LOC112449523 | Bos taurus uncharacterized | 0.007 |
| GCSAML | Germinal center-associated signaling and motility-like protein isoform X2 | 0.007 |
| LOC506408 | Zinc finger protein 786 isoform X2 | 0.007 |
| MGAM | Maltase-glucoamylase, intestinal isoform X2 | 0.007 |
| SNRK | SNF-related serine/threonine-protein kinase isoform X3 | 0.007 |
| GPR82 | Probable G-protein coupled receptor 82 | 0.007 |
| HIP1R | Huntingtin-interacting protein 1-related protein isoform X1 | 0.007 |
| LOC100294723 | Killer cell lectin-like receptor subfamily F member 1 isoform X1 | 0.007 |
| ITGB5 | Integrin beta-5 precursor | 0.007 |
| MIER2 | Mesoderm induction early response protein | 0.007 |
| LOC101906342 | Bos taurus uncharacterized | 0.007 |
| LOC112447744 | Bos taurus uncharacterized | 0.007 |
| DENND4B | DENN domain-containing protein 4B isoform X1 | 0.007 |
