# Supplementary material for: Peripheral leukocyte transcriptomic changes in preweaned Holstein heifer calves with varying stages of Bovine Respiratory Disease
Source: PLoS One. 2026 May 14;21(5):e0349348. doi: 10.1371/journal.pone.0349348 (PMC13175367; doi:10.1371/journal.pone.0349348)
Supplement: S6 Table — (DOCX) [file pone.0349348.s006.docx]

**S6 Table. Descriptive summary table of random forest gene features (n = 38) identified for *Healthy* vs *Chronic*.**

| Gene Symbol | Description | Importance Score |
| --- | --- | --- |
| ABCA9 | ATP-binding cassette sub-family A member 9 | 0.060 |
| MTMR14 | Phosphatidylinositol-3,5-bisphosphate 3-phosphatase MTMR14 | 0.060 |
| ZSWIM4 | Zinc finger SWIM domain-containing protein 4 isoform X1 | 0.040 |
| SALL2 | Sal-like protein 2 isoform X2 | 0.040 |
| SLC41A2 | Solute carrier family 41 member 2 isoform X1 | 0.040 |
| POLD3 | DNA polymerase delta subunit 3 isoform X2 | 0.040 |
| USB1 | U6 snRNA phosphodiesterase isoform X1 | 0.040 |
| NAP1L4 | Nucleosome assembly protein 1-like 4 isoform X3 | 0.040 |
| LOC112445040 | Bos taurus uncharacterized | 0.040 |
| SEC31A | Protein transport protein Sec31A isoform X15 | 0.040 |
| ADGRL1 | Adhesion G protein-coupled receptor L1 isoform X3 | 0.020 |
| JAK1 | Tyrosine-protein kinase JAK1 isoform X1 | 0.020 |
| CFAP298 | Cilia- and flagella-associated protein 298 | 0.020 |
| ANAPC15 | Anaphase-promoting complex subunit 15 isoform X3 | 0.020 |
| LOC112449523 | Bos taurus uncharacterized | 0.020 |
| LOC112441821 | Bos taurus uncharacterized isoform X2 | 0.020 |
| LOC112449553 | Serine/arginine repetitive matrix protein 1-like | 0.020 |
| PGS1 | Bos taurus phosphatidylglycerophosphate synthase 1 isoform X20 | 0.020 |
| CDC27 | Cell division cycle protein 27 homolog isoform X2 | 0.020 |
| AFMID | Kynurenine formamidase isoform X4 | 0.020 |
| ASPHD2 | Aspartate beta-hydroxylase domain-containing protein 2 | 0.020 |
| LOC783362 | Bos taurus uncharacterized isoform X2 | 0.020 |
| LOC618367 | Bos taurus uncharacterized | 0.020 |
| RBM4B | RNA-binding protein 4B isoform X1 | 0.020 |
| P2RX5 | P2X purinoceptor 5 isoform X2 | 0.020 |
| GPAM | Glycerol-3-phosphate acyltransferase 1, mitochondrial | 0.020 |
| CCP110 | Centriolar coiled-coil protein of 110 kDa isoform X1 | 0.020 |
| ZNF839 | Zinc finger protein 839 isoform X2 | 0.020 |
| SRRM2 | Serine/arginine repetitive matrix protein 2 isoform X7 | 0.020 |
| THNSL1 | Threonine synthase-like 1 | 0.020 |
| CASZ1 | Zinc finger protein castor homolog 1 isoform X1 | 0.020 |
| ARL11 | ADP-ribosylation factor-like protein 11 | 0.020 |
| GGTA1 | N-acetyllactosaminide alpha-1,3-galactosyltransferase isoform X5 | 0.020 |
| TIGIT | T-cell immunoreceptor with Ig and ITIM domains isoform X3 | 0.020 |
| SPEG | Striated muscle preferentially expressed protein kinase | 0.020 |
| TRIP12 | E3 ubiquitin-protein ligase TRIP12 isoform X20 | 0.020 |
| CLPTM1 | Putative lipid scramblase CLPTM1 | 0.020 |
| PSMD14 | 26S proteasome non-ATPase regulatory subunit 14 isoform X1 | 0.020 |
