# Supplementary material for: Peripheral leukocyte transcriptomic changes in preweaned Holstein heifer calves with varying stages of Bovine Respiratory Disease
Source: PLoS One. 2026 May 14;21(5):e0349348. doi: 10.1371/journal.pone.0349348 (PMC13175367; doi:10.1371/journal.pone.0349348)
Supplement: S7 Table — (DOCX) [file pone.0349348.s007.docx]

**S7 Table. Descriptive summary table of random forest gene features (n = 57) identified for *Healthy* vs *Resolved*.**

| Gene Symbol | Description | Importance Score |
| --- | --- | --- |
| MXRA5 | Matrix-remodeling-associated protein 5 isoform X1 | 0.214 |
| PPP4R1 | Serine/threonine-protein phosphatase 4 regulatory subunit 1 isoform X5 | 0.116 |
| AMZ2 | Archaemetzincin-2 isoform X1 | 0.056 |
| ZNF354C | Zinc finger protein 354C isoform X2 | 0.043 |
| PRDM10 | PR domain zinc finger protein 10 isoform X1 | 0.041 |
| ZNF746 | Zinc finger protein 746 isoform X4 | 0.037 |
| HNRNPDL | Heterogeneous nuclear ribonucleoprotein Dlike isoform X8 | 0.034 |
| B3GNT7 | UDP-GlcNAc:betaGal beta-1,3-N-acetylglucosaminyltransferase 7 | 0.031 |
| LOC616254 | Intercellular adhesion molecule 2 isoform X9 | 0.024 |
| ATMIN | ATM interactor | 0.023 |
| GLS2 | Glutaminase liver isoform, mitochondrial isoform X3 | 0.021 |
| FAM84B | Protein FAM84B isoform X1 | 0.020 |
| LOC107132340 | Bos taurus uncharacterized isoform X7 | 0.020 |
| B3GNTL1 | UDP-GlcNAc:betaGal beta-1,3-N-acetylglucosaminyltransferase like 1 isoform X3 | 0.020 |
| SLC41A2 | Solute carrier family 41 member 2 isoform X1 | 0.019 |
| KMO | Kynurenine 3-monooxygenase | 0.019 |
| EBI3 | Interleukin-27 subunit beta precursor | 0.018 |
| WDR11 | WD repeat domain 11 isoform X3 | 0.017 |
| H2AFY | Core histone macro-H2A.1 isoform X1 | 0.017 |
| MARK3 | MAP/microtubule affinity-regulating kinase 3 isoform X1 | 0.017 |
| PISD | Phosphatidylserine decarboxylase proenzyme, mitochondrial isoform X5 | 0.016 |
| ANLN | Anillin isoform X10 | 0.016 |
| ZFP90 | Zinc finger protein 90 homolog isoform X1 | 0.016 |
| RPS6KA3 | Ribosomal protein S6 kinase alpha-3 | 0.016 |
| RTKN | Rhotekin isoform X4 | 0.014 |
| ZNF592 | Zinc finger protein 592 | 0.014 |
| GCNA | Acidic repeat-containing protein | 0.011 |
| SCIN | Scinderin | 0.009 |
| ZBTB45 | Zinc finger and BTB domain-containing protein 45 isoform X2 | 0.007 |
| MGAT4A | Alpha-1,3-mannosyl-glycoprotein 4-beta-N-acetylglucosaminyltransferase A isoform X2 | 0.006 |
| GDAP1 | Ganglioside-induced differentiation-associated protein 1 | 0.006 |
| CCNI | Cyclin-I isoform X1 | 0.006 |
| LDLRAD3 | Low-density lipoprotein receptor class A domain-containing protein 3 isoform X3 | 0.005 |
| FITM1 | Fat storage-inducing transmembrane protein 1 isoform X1 | 0.004 |
| DTX1 | E3 ubiquitin-protein ligase DTX1 | 0.004 |
| FARP2 | FERM, ARHGEF and pleckstrin domain-containing protein 2 isoform X5 | 0.003 |
| POGK | Pogo transposable element with KRAB domain isoform X3 | 0.003 |
| C28H10orf105 | Uncharacterized protein C10orf105 homolog | 0.003 |
| LOC101901983 | Bos taurus uncharacterized isoform X2 | 0.003 |
| LOC787234 | Bos taurus uncharacterized isoform | 0.003 |
| PDGFB | Platelet-derived growth factor subunit B isoform X1 | 0.003 |
| LEPROT | Leptin receptor gene-related protein | 0.003 |
| LOC618367 | Bos taurus uncharacterized | 0.003 |
| VDAC1 | Voltage-dependent anion-selective channel protein 1 isoform X1 | 0.003 |
| FIZ1 | Flt3-interacting zinc finger protein 1 isoform X3 | 0.003 |
| PIK3R5 | Phosphoinositide 3-kinase regulatory subunit 5 isoform X3 | 0.002 |
| ELAC2 | Zinc phosphodiesterase ELAC protein 2 | 0.002 |
| IRF2BP1 | Interferon regulatory factor 2-binding protein 1 | 0.002 |
| LOC112442999 | Bos taurus uncharacterized isoform X1 | 0.001 |
| TADA2B | Transcriptional adapter 2-beta | 0.001 |
| TMUB2 | Transmembrane and ubiquitin-like domain-containing protein 2 isoform X2 | 0.001 |
| TICAM2 | TIR domain-containing adapter molecule 2 | 0.001 |
| DOCK10 | Dedicator of cytokinesis protein 10 isoform X7 | 0.001 |
| CLDN3 | Claudin-3 | 0.001 |
| TAL1 | T-cell acute lymphocytic leukemia protein 1 isoform X1 | 0.001 |
| RICTOR | Rapamycin-insensitive companion of mTOR isoform X4 | 0.001 |
| UPF1 | Regulator of nonsense transcripts 1 isoform X1 | 0.001 |
