# Supplementary material for: Peripheral leukocyte transcriptomic changes in preweaned Holstein heifer calves with varying stages of Bovine Respiratory Disease
Source: PLoS One. 2026 May 14;21(5):e0349348. doi: 10.1371/journal.pone.0349348 (PMC13175367; doi:10.1371/journal.pone.0349348)
Supplement: S8 Table — (DOCX) [file pone.0349348.s008.docx]

**S8 Table. Descriptive summary table of significantly enriched terms for *Healthy* vs *Onset*.**

| Module | Vocabulary | Term | Name | Module Size ^a^ | Count In Module ^b^ | Count In Background ^c^ | Fishers ^d^ | Bonferroni ^e^ | Benjamini ^f^ |
| --- | --- | --- | --- | --- | --- | --- | --- | --- | --- |
| Down (M1) | GO: CC | GO:0005663 | DNA replication factor C complex | 6 | 1 | 5 | 0.002 | 0.346 | 0.004 |
| Down (M1) | GO: MF | GO:0003689 | DNA clamp loader activity | 6 | 1 | 9 | 0.002 | 0.369 | 0.004 |
| Down (M1) | IPR | IPR004054 | Potassium channel, voltage dependent, Kv4.1 | 6 | 1 | 4 | 0.001 | 0.147 | 0.003 |
| Down (M1) | IPR | IPR012178 | Replication factor C subunit 1 | 6 | 1 | 5 | 0.001 | 0.177 | 0.003 |
| Down (M1) | IPR | IPR013725 | DNA replication factor RFC1, C-terminal | 6 | 1 | 5 | 0.001 | 0.177 | 0.003 |
| Down (M1) | IPR | IPR003975 | Potassium channel, voltage dependent, Kv4 | 6 | 1 | 7 | 0.001 | 0.235 | 0.003 |
| Down (M1) | IPR | IPR024587 | Potassium channel, voltage dependent, Kv4, C-terminal | 6 | 1 | 7 | 0.001 | 0.235 | 0.003 |
| Down (M1) | IPR | IPR033841 | USP48, peptidase domain | 6 | 1 | 10 | 0.001 | 0.324 | 0.004 |
| Down (M1) | IPR | IPR008921 | DNA polymerase III, clamp loader complex, gamma/delta/delta subunit, C-terminal | 6 | 1 | 16 | 0.002 | 0.500 | 0.005 |
| Down (M1) | IPR | IPR024678 | Serine/threonine-protein kinase OSR1/WNK, CCT domain | 6 | 1 | 44 | 0.006 | 1.000 | 0.008 |
| Down (M1) | IPR | IPR006615 | Peptidase C19, ubiquitin-specific peptidase, DUSP domain | 6 | 1 | 45 | 0.006 | 1.000 | 0.008 |
| Down (M1) | IPR | IPR035927 | DUSP-like superfamily | 6 | 1 | 45 | 0.006 | 1.000 | 0.008 |
| Down (M1) | IPR | IPR003968 | Potassium channel, voltage dependent, Kv | 6 | 1 | 49 | 0.007 | 1.000 | 0.008 |
| Down (M1) | IPR | IPR008422 | KN homeodomain | 6 | 1 | 69 | 0.009 | 1.000 | 0.009 |
| Down (M1) | KEGG | K19553 | TGIF2; homeobox protein TGIF2 | 6 | 1 | 6 | < 0.001 | 0.038 | 0.001 |
| Down (M2) | IPR | IPR006515 | Polyadenylate binding protein, human types 1, 2, 3, 4 | 4 | 1 | 17 | 0.002 | 0.494 | 0.005 |
| Down (M2) | IPR | IPR002004 | Polyadenylate-binding protein/Hyperplastic disc protein, C-terminal | 4 | 1 | 18 | 0.002 | 0.521 | 0.005 |
| Down (M2) | IPR | IPR010508 | Neurobeachin-like, DUF1088 | 4 | 1 | 18 | 0.002 | 0.521 | 0.005 |
| Down (M2) | IPR | IPR036053 | PABC (PABP) domain | 4 | 1 | 18 | 0.002 | 0.521 | 0.005 |
| Down (M2) | IPR | IPR014836 | Integrin beta subunit, cytoplasmic domain | 4 | 1 | 23 | 0.003 | 0.658 | 0.006 |
| Down (M2) | IPR | IPR031570 | Neurobeachin/BDCP, DUF4704 | 4 | 1 | 24 | 0.003 | 0.686 | 0.006 |
| Down (M2) | IPR | IPR040622 | Integrin beta, epidermal growth factor-like domain 1 | 4 | 1 | 25 | 0.003 | 0.713 | 0.006 |
| Down (M2) | IPR | IPR033760 | Integrin beta N-terminal | 4 | 1 | 28 | 0.004 | 0.795 | 0.006 |
| Down (M2) | IPR | IPR012896 | Integrin beta subunit, tail | 4 | 1 | 30 | 0.004 | 0.850 | 0.006 |
| Down (M2) | IPR | IPR036349 | Integrin beta tail domain superfamily | 4 | 1 | 30 | 0.004 | 0.850 | 0.006 |
| Down (M2) | IPR | IPR002369 | Integrin beta subunit, VWA domain | 4 | 1 | 33 | 0.004 | 0.932 | 0.006 |
| Down (M2) | IPR | IPR015812 | Integrin beta subunit | 4 | 1 | 33 | 0.004 | 0.932 | 0.006 |
| Down (M2) | IPR | IPR000409 | BEACH domain | 4 | 1 | 40 | 0.005 | 1.000 | 0.007 |
| Down (M2) | IPR | IPR023362 | PH-BEACH domain | 4 | 1 | 40 | 0.005 | 1.000 | 0.007 |
| Down (M2) | IPR | IPR036372 | BEACH domain superfamily | 4 | 1 | 40 | 0.005 | 1.000 | 0.007 |
| Down (M2) | IPR | IPR007237 | CD20-like, transmembrane domain | 4 | 1 | 50 | 0.006 | 1.000 | 0.008 |
| Down (M2) | IPR | IPR032695 | Integrin domain superfamily | 4 | 1 | 70 | 0.009 | 1.000 | 0.009 |
| Down (M2) | KEGG | K06588 | ITGB5; integrin beta 5 | 4 | 1 | 1 | < 0.001 | 0.032 | 0.001 |
| Down (M2) | KEGG | K22192 | MS4A14; membrane-spanning 4-domains subfamily A member 14 | 4 | 1 | 1 | < 0.001 | 0.032 | 0.001 |
| Down (M2) | KEGG | K24183 | NBEA; neurobeachin | 4 | 1 | 9 | 0.001 | 0.161 | 0.003 |
| Down (M3) | GO: BP | GO:0042998 | Positive regulation of Golgi to plasma membrane protein transport | 3 | 1 | 5 | < 0.001 | 0.080 | 0.002 |
| Down (M3) | GO: CC | GO:0005802 | Trans-Golgi network | 3 | 1 | 11 | 0.002 | 0.462 | 0.005 |
| Down (M3) | GO: MF | GO:0071253 | connexin binding | 3 | 1 | 5 | < 0.001 | 0.040 | 0.001 |
| Down (M3) | IPR | IPR028129 | Consortin, C-terminal domain | 3 | 1 | 5 | < 0.001 | 0.018 | 0.001 |
| Down (M3) | IPR | IPR002126 | Cadherin-like | 3 | 1 | 232 | 0.003 | 0.685 | 0.006 |
| Down (M3) | IPR | IPR015919 | Cadherin-like superfamily | 3 | 1 | 240 | 0.003 | 0.709 | 0.006 |
| Down (M3) | KEGG | K17581 | CNST; consortin | 3 | 1 | 5 | < 0.001 | 0.032 | 0.001 |
| Down (M4) | IPR | IPR006561 | DZF domain | 2 | 1 | 26 | 0.001 | 0.185 | 0.003 |
| Down (M4) | IPR | IPR003137 | PA domain | 2 | 1 | 44 | 0.001 | 0.309 | 0.004 |
| Down (M4) | IPR | IPR003604 | Matrin/U1-C-like, C2H2-type zinc finger | 2 | 1 | 121 | 0.004 | 0.837 | 0.006 |
| Down (M4) | KEGG | K13203 | ZFR; zinc finger RNA-binding protein | 2 | 1 | 7 | < 0.001 | 0.043 | 0.001 |
| Up (M1) | GO: MF | GO:0005509 | Calcium ion binding | 115 | 9 | 1545 | 0.001 | 0.312 | 0.004 |
| Up (M1) | GO: MF | GO:0005262 | Calcium channel activity | 115 | 2 | 42 | 0.003 | 0.582 | 0.006 |
| Up (M1) | GO: CC | GO:0016020 | Membrane | 115 | 11 | 3304 | 0.007 | 1.000 | 0.008 |
| Up (M1) | GO: BP | GO:0006691 | Leukotriene metabolic process | 115 | 1 | 3 | 0.008 | 1.000 | 0.009 |
| Up (M1) | GO: MF | GO:0004089 | Carbonate dehydratase activity | 115 | 1 | 4 | 0.008 | 1.000 | 0.009 |
| Up (M1) | GO: BP | GO:1901642 | Nucleoside transmembrane transport | 115 | 3 | 27 | < 0.001 | 0.006 | < 0.001 |
| Up (M1) | GO: MF | GO:0004908 | Interleukin-1 receptor activity | 115 | 4 | 42 | < 0.001 | < 0.001 | < 0.001 |
| Up (M1) | GO: MF | GO:0005337 | Nucleoside transmembrane transporter activity | 115 | 3 | 27 | < 0.001 | 0.004 | < 0.001 |
| Up (M1) | GO: MF | GO:0004910 | Interleukin-1, type II, blocking receptor activity | 115 | 2 | 7 | < 0.001 | 0.023 | 0.001 |
| Up (M1) | GO: MF | GO:0016791 | phosphatase activity | 115 | 5 | 447 | 0.001 | 0.246 | 0.003 |
| Up (M1) | IPR | IPR009449 | GDP/GTP exchange factor Sec2, N-terminal | 115 | 5 | 20 | < 0.001 | < 0.001 | < 0.001 |
| Up (M1) | IPR | IPR001952 | Alkaline phosphatase | 115 | 4 | 13 | < 0.001 | < 0.001 | < 0.001 |
| Up (M1) | IPR | IPR035875 | BMX, SH2 domain | 115 | 3 | 4 | < 0.001 | < 0.001 | < 0.001 |
| Up (M1) | IPR | IPR013787 | S100/CaBP-9k-type, calcium binding, subdomain | 115 | 4 | 35 | < 0.001 | < 0.001 | < 0.001 |
| Up (M1) | IPR | IPR002668 | Concentrative nucleoside transporter N-terminal domain | 115 | 3 | 8 | < 0.001 | < 0.001 | < 0.001 |
| Up (M1) | IPR | IPR011642 | Nucleoside transporter/FeoB GTPase, Gate domain | 115 | 3 | 8 | < 0.001 | < 0.001 | < 0.001 |
| Up (M1) | IPR | IPR011657 | Concentrative nucleoside transporter C-terminal domain | 115 | 3 | 8 | < 0.001 | < 0.001 | < 0.001 |
| Up (M1) | IPR | IPR018270 | Concentrative nucleoside transporter, metazoan/bacterial | 115 | 3 | 8 | < 0.001 | < 0.001 | < 0.001 |
| Up (M1) | IPR | IPR004074 | Interleukin-1 receptor type I/II | 115 | 4 | 42 | < 0.001 | < 0.001 | < 0.001 |
| Up (M1) | IPR | IPR001245 | Serine-threonine/tyrosine-protein kinase, catalytic domain | 115 | 8 | 507 | < 0.001 | 0.001 | < 0.001 |
| Up (M1) | IPR | IPR004156 | Organic anion transporter polypeptide | 115 | 3 | 23 | < 0.001 | 0.003 | < 0.001 |
| Up (M1) | IPR | IPR001562 | Zinc finger, Btk motif | 115 | 3 | 25 | < 0.001 | 0.004 | < 0.001 |
| Up (M1) | IPR | IPR003087 | Neutrophil gelatinase-associated lipocalin/epididymal-specific lipocalin-12 | 115 | 2 | 3 | < 0.001 | 0.007 | < 0.001 |
| Up (M1) | IPR | IPR015321 | Type I cytokine receptor, cytokine-binding domain | 115 | 3 | 31 | < 0.001 | 0.007 | < 0.001 |
| Up (M1) | IPR | IPR037768 | Copine, C2B domain | 115 | 3 | 33 | < 0.001 | 0.008 | < 0.001 |
| Up (M1) | IPR | IPR010734 | Copine, C-terminal | 115 | 3 | 37 | < 0.001 | 0.011 | < 0.001 |
| Up (M1) | IPR | IPR017850 | Alkaline-phosphatase-like, core domain superfamily | 115 | 4 | 120 | < 0.001 | 0.017 | 0.001 |
| Up (M1) | IPR | IPR041416 | IL-1Ra-like, immunoglobulin domain | 115 | 3 | 45 | < 0.001 | 0.020 | 0.001 |
| Up (M1) | IPR | IPR004077 | Interleukin-1 receptor type II | 115 | 2 | 7 | < 0.001 | 0.025 | 0.001 |
| Up (M1) | IPR | IPR002048 | EF-hand domain | 115 | 7 | 733 | < 0.001 | 0.084 | 0.002 |
| Up (M1) | IPR | IPR040907 | IL-3 receptor alpha chain, N-terminal | 115 | 2 | 15 | < 0.001 | 0.092 | 0.002 |
| Up (M1) | IPR | IPR002035 | von Willebrand factor, type A | 115 | 4 | 204 | 0.001 | 0.119 | 0.003 |
| Up (M1) | IPR | IPR001781 | Zinc finger, LIM-type | 115 | 5 | 390 | 0.001 | 0.161 | 0.003 |
| Up (M1) | IPR | IPR001478 | PDZ domain | 115 | 7 | 836 | 0.001 | 0.180 | 0.003 |
| Up (M1) | IPR | IPR036058 | Kazal domain superfamily | 115 | 3 | 102 | 0.001 | 0.197 | 0.003 |
| Up (M1) | IPR | IPR036034 | PDZ superfamily | 115 | 7 | 856 | 0.001 | 0.207 | 0.003 |
| Up (M1) | IPR | IPR002350 | Kazal domain | 115 | 3 | 106 | 0.001 | 0.219 | 0.003 |
| Up (M1) | IPR | IPR011992 | EF-hand domain pair | 115 | 7 | 928 | 0.001 | 0.326 | 0.004 |
| Up (M1) | IPR | IPR036465 | von Willebrand factor A-like domain superfamily | 115 | 4 | 294 | 0.002 | 0.447 | 0.005 |
| Up (M1) | IPR | IPR001740 | GPCR family 2, EMR1-like receptor | 115 | 2 | 36 | 0.002 | 0.463 | 0.005 |
| Up (M1) | IPR | IPR005461 | Transient receptor potential channel, canonical 5 | 115 | 1 | 1 | 0.003 | 0.787 | 0.006 |
| Up (M1) | IPR | IPR006970 | PT repeat | 115 | 1 | 1 | 0.003 | 0.787 | 0.006 |
| Up (M1) | IPR | IPR013740 | Redoxin | 115 | 1 | 1 | 0.003 | 0.787 | 0.006 |
| Up (M1) | IPR | IPR028029 | Signal transducer CD24 | 115 | 1 | 1 | 0.003 | 0.787 | 0.006 |
| Up (M1) | IPR | IPR037944 | Peroxiredoxin-5-like | 115 | 1 | 1 | 0.003 | 0.787 | 0.006 |
| Up (M1) | IPR | IPR003303 | Filaggrin | 115 | 1 | 2 | 0.005 | 1.000 | 0.007 |
| Up (M1) | IPR | IPR027954 | Transcobalamin-like, C-terminal domain | 115 | 1 | 2 | 0.005 | 1.000 | 0.007 |
| Up (M1) | IPR | IPR000566 | Lipocalin/cytosolic fatty-acid binding domain | 115 | 2 | 60 | 0.005 | 1.000 | 0.007 |
| Up (M1) | IPR | IPR000832 | GPCR, family 2, secretin-like | 115 | 3 | 213 | 0.007 | 1.000 | 0.008 |
| Up (M1) | IPR | IPR000436 | Sushi/SCR/CCP domain | 115 | 3 | 216 | 0.007 | 1.000 | 0.008 |
| Up (M1) | IPR | IPR035976 | Sushi/SCR/CCP superfamily | 115 | 3 | 216 | 0.007 | 1.000 | 0.008 |
| Up (M1) | IPR | IPR001446 | 5-lipoxygenase-activating protein | 115 | 1 | 3 | 0.007 | 1.000 | 0.008 |
| Up (M1) | IPR | IPR041874 | Carbonic anhydrase, CA4/CA15 | 115 | 1 | 3 | 0.007 | 1.000 | 0.008 |
| Up (M1) | IPR | IPR042153 | VDR, DNA-binding domain | 115 | 1 | 3 | 0.007 | 1.000 | 0.008 |
| Up (M1) | IPR | IPR012674 | Calycin | 115 | 2 | 69 | 0.007 | 1.000 | 0.008 |
| Up (M1) | IPR | IPR017981 | GPCR, family 2-like, 7TM | 115 | 3 | 230 | 0.008 | 1.000 | 0.009 |
| Up (M1) | IPR | IPR013783 | Immunoglobulin-like fold | 115 | 11 | 2665 | 0.008 | 1.000 | 0.009 |
| Up (M1) | IPR | IPR000324 | Vitamin D receptor | 115 | 1 | 4 | 0.009 | 1.000 | 0.009 |
| Up (M1) | IPR | IPR002435 | Sodium:neurotransmitter symporter, noradrenaline | 115 | 1 | 4 | 0.009 | 1.000 | 0.009 |
| Up (M1) | IPR | IPR010533 | Vertebrate interleukin-3 regulated transcription factor | 115 | 1 | 4 | 0.009 | 1.000 | 0.009 |
| Up (M1) | IPR | IPR016743 | Nuclear factor interleukin-3-regulated protein | 115 | 1 | 4 | 0.009 | 1.000 | 0.009 |
| Up (M1) | IPR | IPR017076 | Kremen | 115 | 1 | 4 | 0.009 | 1.000 | 0.009 |
| Up (M1) | IPR | IPR013151 | Immunoglobulin-like beta-sandwich domain | 115 | 3 | 237 | 0.009 | 1.000 | 0.009 |
| Up (M1) | IPR | IPR013806 | Kringle-like fold | 115 | 2 | 82 | 0.010 | 1.000 | 0.010 |
| Up (M1) | KEGG | K01077 | E3.1.3.1, phoA, phoB; alkaline phosphatase [EC:3.1.3.1] | 115 | 4 | 13 | < 0.001 | < 0.001 | < 0.001 |
| Up (M1) | KEGG | K08896 | BMX, ETK; cytoplasmic tyrosine-protein kinase BMX [EC:2.7.10.2] | 115 | 3 | 4 | < 0.001 | < 0.001 | < 0.001 |
| Up (M1) | KEGG | K21128 | S100A9; protein S100-A9 | 115 | 3 | 4 | < 0.001 | < 0.001 | < 0.001 |
| Up (M1) | KEGG | K11536 | SLC28A; pyrimidine nucleoside transport protein | 115 | 3 | 6 | < 0.001 | < 0.001 | < 0.001 |
| Up (M1) | KEGG | K16779 | RAB3IP, RABIN8; Rab-3A-interacting protein | 115 | 3 | 16 | < 0.001 | 0.001 | < 0.001 |
| Up (M1) | KEGG | K04387 | IL1R2, CD121b; interleukin 1 receptor type II | 115 | 2 | 2 | < 0.001 | 0.004 | < 0.001 |
| Up (M1) | KEGG | K14355 | SLCO4C; solute carrier organic anion transporter family, member 4C | 115 | 2 | 2 | < 0.001 | 0.004 | < 0.001 |
| Up (M1) | KEGG | K21129 | LCN2; lipocalin 2 | 115 | 2 | 2 | < 0.001 | 0.004 | < 0.001 |
| Up (M1) | KEGG | K05744 | LIMK2; LIM domain kinase 2 [EC:2.7.11.1] | 115 | 2 | 4 | < 0.001 | 0.009 | < 0.001 |
| Up (M1) | KEGG | K24524 | CPNE1_2_3; copine 1/2/3 | 115 | 2 | 5 | < 0.001 | 0.013 | < 0.001 |
| Up (M1) | KEGG | K01335 | CFB; complement factor B [EC:3.4.21.47] | 115 | 1 | 1 | 0.003 | 0.749 | 0.006 |
| Up (M1) | KEGG | K01403 | MMP9; matrix metalloproteinase-9 (gelatinase B) [EC:3.4.24.35] | 115 | 1 | 1 | 0.003 | 0.749 | 0.006 |
| Up (M1) | KEGG | K04685 | CDKN2B, P15, INK4B; cyclin-dependent kinase inhibitor 2B | 115 | 1 | 1 | 0.003 | 0.749 | 0.006 |
| Up (M1) | KEGG | K04737 | IL3RA, CD123; interleukin 3 receptor alpha | 115 | 1 | 1 | 0.003 | 0.749 | 0.006 |
| Up (M1) | KEGG | K04968 | TRPC5; transient receptor potential cation channel subfamily C member 5 | 115 | 1 | 1 | 0.003 | 0.749 | 0.006 |
| Up (M1) | KEGG | K06469 | CD24; CD24 antigen | 115 | 1 | 1 | 0.003 | 0.749 | 0.006 |
| Up (M1) | KEGG | K09634 | TMPRSS3; transmembrane protease serine 3 [EC:3.4.21.-] | 115 | 1 | 1 | 0.003 | 0.749 | 0.006 |
| Up (M1) | KEGG | K11187 | PRDX5; peroxiredoxin 5 [EC:1.11.1.24] | 115 | 1 | 1 | 0.003 | 0.749 | 0.006 |
| Up (M1) | KEGG | K17400 | DNMT3L; DNA (cytosine-5)-methyltransferase 3-like | 115 | 1 | 1 | 0.003 | 0.749 | 0.006 |
| Up (M1) | KEGG | K18166 | FOXRED1; FAD-dependent oxidoreductase domain-containing protein 1 | 115 | 1 | 1 | 0.003 | 0.749 | 0.006 |
| Up (M1) | KEGG | K18261 | DYSF; dysferlin | 115 | 1 | 1 | 0.003 | 0.749 | 0.006 |
| Up (M1) | KEGG | K20735 | ALOX5AP, FLAP; arachidonate 5-lipoxygenase-activating protein | 115 | 1 | 1 | 0.003 | 0.749 | 0.006 |
| Up (M1) | KEGG | K21127 | S100A8; protein S100-A8 | 115 | 1 | 1 | 0.003 | 0.749 | 0.006 |
| Up (M1) | KEGG | K24334 | MEGF9; multiple epidermal growth factor-like domains protein 9 | 115 | 1 | 1 | 0.003 | 0.749 | 0.006 |
| Up (M1) | KEGG | K25447 | OLFM4; olfactomedin-4 | 115 | 1 | 1 | 0.003 | 0.749 | 0.006 |
| Up (M1) | KEGG | K25565 | IL18BP; interleukin-18-binding protein | 115 | 1 | 1 | 0.003 | 0.749 | 0.006 |
| Up (M1) | KEGG | K25741 | TCN1; transcobalamin-1 | 115 | 1 | 1 | 0.003 | 0.749 | 0.006 |
| Up (M1) | KEGG | K04961 | RYR1; ryanodine receptor 1 | 115 | 1 | 2 | 0.005 | 1.000 | 0.007 |
| Up (M1) | KEGG | K05074 | IL15RA, CD215; interleukin 15 receptor alpha | 115 | 1 | 2 | 0.005 | 1.000 | 0.007 |
| Up (M1) | KEGG | K05173 | IL18R1, IL1RRP, CD218a; interleukin 18 receptor 1 | 115 | 1 | 2 | 0.005 | 1.000 | 0.007 |
| Up (M1) | KEGG | K08452 | ADGRG3, GPR97; adhesion G-protein coupled receptor G3 | 115 | 1 | 2 | 0.005 | 1.000 | 0.007 |
| Up (M1) | KEGG | K10474 | KBTBD6_7; kelch repeat and BTB domain-containing protein 6/7 | 115 | 1 | 2 | 0.005 | 1.000 | 0.007 |
| Up (M1) | KEGG | K14377 | OSCAR; osteoclast-associated immunoglobulin-like receptor | 115 | 1 | 2 | 0.005 | 1.000 | 0.007 |
| Up (M1) | KEGG | K26816 | GLT1D1; glycosyltransferase 1 domain-containing protein 1 [EC:2.4.-.-] | 115 | 1 | 2 | 0.005 | 1.000 | 0.007 |
| Up (M1) | KEGG | K05068 | IL2RA, CD25; interleukin 2 receptor alpha | 115 | 1 | 3 | 0.007 | 1.000 | 0.008 |
| Up (M1) | KEGG | K08539 | VDR, NR1I1; vitamin D3 receptor | 115 | 1 | 3 | 0.007 | 1.000 | 0.008 |
| Up (M1) | KEGG | K14354 | SLCO4A; solute carrier organic anion transporter family, member 4A | 115 | 1 | 3 | 0.007 | 1.000 | 0.008 |
| Up (M1) | KEGG | K16847 | TIAM2; T-lymphoma invasion and metastasis-inducing protein 2 | 115 | 1 | 3 | 0.007 | 1.000 | 0.008 |
| Up (M1) | KEGG | K18018 | PTPN5; tyrosine-protein phosphatase non-receptor type 5 [EC:3.1.3.48] | 115 | 1 | 3 | 0.007 | 1.000 | 0.008 |
| Up (M1) | KEGG | K18246 | CA4; carbonic anhydrase 4 [EC:4.2.1.1] | 115 | 1 | 3 | 0.007 | 1.000 | 0.008 |
| Up (M1) | KEGG | K05035 | SLC6A2, NET; solute carrier family 6 (neurotransmitter transporter, noradrenalin) member 2 | 115 | 1 | 4 | 0.008 | 1.000 | 0.009 |
| Up (M1) | KEGG | K09059 | NFIL3, E4BP4; nuclear factor, interleukin 3 regulated | 115 | 1 | 4 | 0.008 | 1.000 | 0.009 |
| Up (M1) | KEGG | K23091 | KREMEN; kremen protein | 115 | 1 | 4 | 0.008 | 1.000 | 0.009 |
| Up (M1) | KEGG | K01950 | E6.3.5.1, NADSYN1, QNS1, nadE; NAD+ synthase (glutamine-hydrolysing) [EC:6.3.5.1] | 115 | 1 | 5 | 0.010 | 1.000 | 0.010 |
| Up (M1) | KEGG | K05075 | IL21R, CD360; interleukin 21 receptor | 115 | 1 | 5 | 0.010 | 1.000 | 0.010 |
| Up (M1) | KEGG | K17908 | WIPI1_2, ATG18; autophagy-related protein 18 | 115 | 1 | 5 | 0.010 | 1.000 | 0.010 |
| Up (M2) | GO: BP | GO:0007166 | Cell surface receptor signaling pathway | 10 | 2 | 259 | 0.001 | 0.274 | 0.003 |
| Up (M2) | GO: MF | GO:0004888 | Transmembrane signaling receptor activity | 10 | 2 | 321 | 0.003 | 0.599 | 0.006 |
| Up (M2) | IPR | IPR000203 | GPS motif | 10 | 2 | 167 | 0.001 | 0.140 | 0.003 |
| Up (M2) | IPR | IPR000832 | GPCR, family 2, secretin-like | 10 | 2 | 213 | 0.001 | 0.225 | 0.003 |
| Up (M2) | IPR | IPR017981 | GPCR, family 2-like, 7TM | 10 | 2 | 230 | 0.001 | 0.261 | 0.003 |
| Up (M2) | IPR | IPR004074 | Interleukin-1 receptor type I/II | 10 | 1 | 42 | 0.009 | 1.000 | 0.009 |
| Up (M2) | IPR | IPR014837 | EF-hand, Ca insensitive | 10 | 1 | 43 | 0.009 | 1.000 | 0.010 |
| Up (M2) | IPR | IPR041416 | IL-1Ra-like, immunoglobulin domain | 10 | 1 | 45 | 0.010 | 1.000 | 0.010 |
| Up (M2) | KEGG | K04602 | CELSR3; cadherin EGF LAG seven-pass G-type receptor 3 | 10 | 1 | 1 | < 0.001 | 0.075 | 0.002 |
| Up (M2) | KEGG | K08452 | ADGRG3, GPR97; adhesion G-protein coupled receptor G3 | 10 | 1 | 2 | 0.001 | 0.113 | 0.003 |
| Up (M2) | KEGG | K17480 | AATK, LMTK1; lemur tyrosine kinase 1 [EC:2.7.10.1] | 10 | 1 | 3 | 0.001 | 0.150 | 0.003 |
| Up (M2) | KEGG | K25593 | CASP8AP2; CASP8-associated protein 2 | 10 | 1 | 5 | 0.001 | 0.225 | 0.003 |
| Up (M2) | KEGG | K04723 | IL1RAP; interleukin 1 receptor accessory protein | 10 | 1 | 10 | 0.002 | 0.412 | 0.005 |
| Up (M2) | KEGG | K23484 | DNM2; dynamin 2 [EC:3.6.5.5] | 10 | 1 | 15 | 0.003 | 0.600 | 0.006 |
| Up (M2) | KEGG | K05699 | ACTN1_4; actinin alpha 1/4 | 10 | 1 | 16 | 0.003 | 0.637 | 0.006 |
| Up (M3) | GO: MF | GO:0030366 | Molybdopterin synthase activity | 13 | 1 | 4 | 0.001 | 0.151 | 0.003 |
| Up (M3) | GO: CC | GO:0019008 | Obsolete molybdopterin synthase complex | 13 | 1 | 8 | 0.002 | 0.519 | 0.005 |
| Up (M3) | GO: BP | GO:0006777 | Mo-molybdopterin cofactor biosynthetic process | 13 | 1 | 11 | 0.003 | 0.639 | 0.006 |
| Up (M3) | GO: BP | GO:0090026 | Positive regulation of monocyte chemotaxis | 13 | 1 | 12 | 0.003 | 0.692 | 0.006 |
| Up (M3) | GO: CC | GO:0005829 | Cytosol | 13 | 1 | 24 | 0.006 | 1.000 | 0.008 |
| Up (M3) | GO: MF | GO:0016493 | C-C chemokine receptor activity | 13 | 1 | 52 | 0.007 | 1.000 | 0.008 |
| Up (M3) | IPR | IPR003448 | Molybdopterin biosynthesis MoaE | 13 | 1 | 4 | 0.001 | 0.152 | 0.003 |
| Up (M3) | IPR | IPR028888 | Molybdopterin synthase catalytic subunit, eukaryotes | 13 | 1 | 4 | 0.001 | 0.152 | 0.003 |
| Up (M3) | IPR | IPR036563 | Molybdopterin biosynthesis MoaE subunit superfamily | 13 | 1 | 4 | 0.001 | 0.152 | 0.003 |
| Up (M3) | IPR | IPR042575 | Ubiquitin-associated protein 1, C-terminal | 13 | 1 | 6 | 0.001 | 0.213 | 0.003 |
| Up (M3) | IPR | IPR002236 | CC chemokine receptor 1 | 13 | 1 | 8 | 0.001 | 0.274 | 0.003 |
| Up (M3) | IPR | IPR023340 | UMA domain | 13 | 1 | 12 | 0.002 | 0.395 | 0.004 |
| Up (M3) | IPR | IPR012315 | KASH domain | 13 | 1 | 45 | 0.006 | 1.000 | 0.008 |
| Up (M3) | IPR | IPR006011 | Syntaxin, N-terminal domain | 13 | 1 | 62 | 0.008 | 1.000 | 0.009 |
| Up (M3) | KEGG | K24926 | TTC1; tetratricopeptide repeat protein 1 | 13 | 1 | 2 | 0.001 | 0.145 | 0.003 |
| Up (M3) | KEGG | K24629 | UBAP1; ubiquitin-associated protein 1 | 13 | 1 | 3 | 0.001 | 0.193 | 0.003 |
| Up (M3) | KEGG | K03635 | MOCS2B, moaE; molybdopterin synthase catalytic subunit [EC:2.8.1.12] | 13 | 1 | 4 | 0.001 | 0.241 | 0.003 |
| Up (M3) | KEGG | K11170 | DHRSX; polyprenol dehydrogenase [NAD(P)H] [EC:1.1.1.441] | 13 | 1 | 4 | 0.001 | 0.241 | 0.003 |
| Up (M3) | KEGG | K15618 | BCL6; B-cell lymphoma 6 protein | 13 | 1 | 4 | 0.001 | 0.241 | 0.003 |
| Up (M3) | KEGG | K04176 | CCR1, CD191; C-C chemokine receptor type 1 | 13 | 1 | 8 | 0.002 | 0.434 | 0.005 |
| Up (M3) | KEGG | K08487 | STX11; syntaxin 11 | 13 | 1 | 9 | 0.002 | 0.482 | 0.005 |
| Up (M3) | KEGG | K15378 | SLC45A1_2_4; solute carrier family 45, member 1/2/4 | 13 | 1 | 14 | 0.003 | 0.723 | 0.006 |
| Up (M3) | KEGG | K19326 | SYNE1; nesprin-1 | 13 | 1 | 19 | 0.004 | 0.963 | 0.006 |
| Up (M4) | GO: MF | GO:0005283 | Amino acid:sodium symporter activity | 10 | 1 | 13 | 0.002 | 0.517 | 0.005 |
| Up (M4) | GO: CC | GO:0016012 | Sarcoglycan complex | 10 | 1 | 17 | 0.005 | 1.000 | 0.007 |
| Up (M4) | GO: MF | GO:0042578 | Phosphoric ester hydrolase activity | 10 | 1 | 33 | 0.006 | 1.000 | 0.007 |
| Up (M4) | GO: BP | GO:0035556 | Intracellular signal transduction | 10 | 2 | 889 | 0.007 | 1.000 | 0.008 |
| Up (M4) | GO: BP | GO:0006836 | Neurotransmitter transport | 10 | 1 | 46 | 0.007 | 1.000 | 0.008 |
| Up (M4) | GO: BP | GO:0046856 | Phosphatidylinositol dephosphorylation | 10 | 1 | 56 | 0.008 | 1.000 | 0.009 |
| Up (M4) | IPR | IPR035866 | SOCS7, SH2 domain | 10 | 1 | 4 | 0.001 | 0.260 | 0.003 |
| Up (M4) | IPR | IPR037346 | SOCS7, SOCS box domain | 10 | 1 | 4 | 0.001 | 0.260 | 0.003 |
| Up (M4) | IPR | IPR034951 | Regulator of G-protein signalling 3, RGS domain | 10 | 1 | 10 | 0.003 | 0.571 | 0.006 |
| Up (M4) | IPR | IPR008908 | Sarcoglycan alpha/epsilon | 10 | 1 | 11 | 0.003 | 0.623 | 0.006 |
| Up (M4) | IPR | IPR031597 | KELK-motif containing domain | 10 | 1 | 12 | 0.003 | 0.675 | 0.006 |
| Up (M4) | IPR | IPR003028 | Sodium:neurotransmitter symporter, glycine, type 1 | 10 | 1 | 13 | 0.003 | 0.727 | 0.006 |
| Up (M4) | IPR | IPR006644 | Dystroglycan-type cadherin-like | 10 | 1 | 17 | 0.004 | 0.934 | 0.006 |
| Up (M4) | IPR | IPR034971 | Synaptojanin-1, RNA recognition motif | 10 | 1 | 17 | 0.004 | 0.934 | 0.006 |
| Up (M4) | IPR | IPR009449 | GDP/GTP exchange factor Sec2, N-terminal | 10 | 1 | 20 | 0.005 | 1.000 | 0.007 |
| Up (M4) | IPR | IPR004198 | Zinc finger, C5HC2-type | 10 | 1 | 23 | 0.006 | 1.000 | 0.007 |
| Up (M4) | IPR | IPR015047 | Synaptojanin-1/2, RNA recognition motif | 10 | 1 | 25 | 0.006 | 1.000 | 0.008 |
| Up (M4) | IPR | IPR002013 | SAC domain | 10 | 1 | 33 | 0.008 | 1.000 | 0.009 |
| Up (M4) | IPR | IPR014930 | Myotonic dystrophy protein kinase, coiled coil | 10 | 1 | 36 | 0.009 | 1.000 | 0.009 |
| Up (M4) | IPR | IPR003349 | JmjN domain | 10 | 1 | 38 | 0.009 | 1.000 | 0.009 |
| Up (M4) | IPR | IPR022166 | UBAP2/protein lingerer | 10 | 1 | 41 | 0.010 | 1.000 | 0.010 |
| Up (M4) | KEGG | K12565 | SGCA; alpha-sarcoglycan | 10 | 1 | 2 | 0.001 | 0.161 | 0.003 |
| Up (M4) | KEGG | K07524 | RGS3; regulator of G-protein signalling 3 | 10 | 1 | 4 | 0.001 | 0.268 | 0.003 |
| Up (M4) | KEGG | K05038 | SLC6A5_9, GLYT; solute carrier family 6 (neurotransmitter transporter, glycine) member 5/9 | 10 | 1 | 5 | 0.001 | 0.321 | 0.004 |
| Up (M4) | KEGG | K11478 | JARID2, JMJ; protein Jumonji | 10 | 1 | 5 | 0.001 | 0.321 | 0.004 |
| Up (M4) | KEGG | K04699 | SOCS6_7; suppressor of cytokine signaling 6/7 | 10 | 1 | 12 | 0.003 | 0.696 | 0.006 |
| Up (M4) | KEGG | K16779 | RAB3IP, RABIN8; Rab-3A-interacting protein | 10 | 1 | 16 | 0.004 | 0.910 | 0.006 |
| Up (M4) | KEGG | K16307 | CDC42BP; serine/threonine-protein kinase MRCK [EC:2.7.11.1] | 10 | 1 | 18 | 0.005 | 1.000 | 0.007 |
| Up (M4) | KEGG | K24814 | HEATR5, LAA1; HEAT repeat-containing protein 5 | 10 | 1 | 18 | 0.005 | 1.000 | 0.007 |
| Up (M4) | KEGG | K20279 | SYNJ; synaptojanin [EC:3.1.3.36] | 10 | 1 | 23 | 0.006 | 1.000 | 0.008 |
| Up (M4) | KEGG | K26545 | UBAP2; ubiquitin-associated protein 2 | 10 | 1 | 37 | 0.009 | 1.000 | 0.009 |

These terms were identified using the program FUNC-E, a python package for the functional enrichment analysis of gene sets.

^a^ Module size: The total number of genes in the module.

^b^ Count in Module: The number of genes in the module annotated with the term.

^c^ Count in Background: The total number of genes in the background annotated with the term.

^d^ Fisher’s p-value: The raw p-value from the Fisher’s exact test.

^e^ Bonferroni: The Bonferroni-corrected p-value for multiple testing

^f^ Benjamini: The Benjamini-Hochberg (FDR) corrected p-value for multiple testing.
