# Supplementary material for: Peripheral leukocyte transcriptomic changes in preweaned Holstein heifer calves with varying stages of Bovine Respiratory Disease
Source: PLoS One. 2026 May 14;21(5):e0349348. doi: 10.1371/journal.pone.0349348 (PMC13175367; doi:10.1371/journal.pone.0349348)
Supplement: S9 Table — (DOCX) [file pone.0349348.s009.docx]

**S9 Table. Descriptive summary table of significantly enriched terms for *Healthy* vs *Chronic*.**

| Module | Vocabulary | Term | Name | Module Size ^a^ | Count In Module ^b^ | Count In Background ^c^ | Fishers ^d^ | Bonferroni ^e^ | Benjamini ^f^ |
| --- | --- | --- | --- | --- | --- | --- | --- | --- | --- |
| Down (M1) | KEGG | K09551 | CLGN; calmegin | 2 | 1 | 3 | < 0.001 | 0.010 | 0.002 |
| Down (M1) | IPR | IPR009033 | Calreticulin/calnexin, P domain superfamily | 2 | 1 | 8 | < 0.001 | 0.024 | 0.002 |
| Down (M1) | IPR | IPR001580 | Calreticulin/calnexin | 2 | 1 | 9 | < 0.001 | 0.026 | 0.002 |
| Down (M1) | KEGG | K06114 | SPTAN; spectrin alpha, non-erythrocytic (fodrin) | 2 | 1 | 21 | 0.001 | 0.058 | 0.003 |
| Down (M1) | IPR | IPR035825 | Alpha Spectrin, SH3 domain | 2 | 1 | 21 | 0.001 | 0.058 | 0.003 |
| Down (M1) | IPR | IPR014837 | EF-hand, Ca insensitive | 2 | 1 | 43 | 0.002 | 0.116 | 0.003 |
| Down (M1) | GO: BP | GO:0006457 | Protein folding | 2 | 1 | 80 | 0.002 | 0.132 | 0.004 |
| Down (M1) | GO: MF | GO:0005509 | Calcium ion binding | 2 | 2 | 1545 | 0.005 | 0.280 | 0.006 |
| Down (M1) | GO: MF | GO:0051082 | Unfolded protein binding | 2 | 1 | 72 | 0.005 | 0.299 | 0.006 |
| Up (M1) | KEGG | K12365 | PREX1; phosphatidylinositol-3,4,5-trisphosphate-dependent Rac exchanger 1 protein | 1 | 1 | 2 | < 0.001 | 0.004 | 0.002 |
| Up (M1) | IPR | IPR000591 | DEP domain | 1 | 1 | 128 | 0.005 | 0.278 | 0.006 |
| Up (M2) | IPR | IPR015321 | Type I cytokine receptor, cytokine-binding domain | 20 | 2 | 31 | < 0.001 | 0.002 | 0.002 |
| Up (M2) | KEGG | K04685 | CDKN2B, P15, INK4B; cyclin-dependent kinase inhibitor 2B | 20 | 1 | 1 | < 0.001 | 0.024 | 0.002 |
| Up (M2) | KEGG | K04737 | IL3RA, CD123; interleukin 3 receptor alpha | 20 | 1 | 1 | < 0.001 | 0.024 | 0.002 |
| Up (M2) | KEGG | K04387 | IL1R2, CD121b; interleukin 1 receptor type II | 20 | 1 | 2 | 0.001 | 0.035 | 0.002 |
| Up (M2) | KEGG | K04961 | RYR1; ryanodine receptor 1 | 20 | 1 | 2 | 0.001 | 0.035 | 0.002 |
| Up (M2) | KEGG | K19821 | SERPINB2, PAI2; plasminogen activator inhibitor 2 | 20 | 1 | 2 | 0.001 | 0.035 | 0.002 |
| Up (M2) | KEGG | K20394 | SESN2; sestrin 2 | 20 | 1 | 2 | 0.001 | 0.035 | 0.002 |
| Up (M2) | KEGG | K05068 | IL2RA, CD25; interleukin 2 receptor alpha | 20 | 1 | 3 | 0.001 | 0.047 | 0.003 |
| Up (M2) | KEGG | K18246 | CA4; carbonic anhydrase 4 [EC:4.2.1.1] | 20 | 1 | 3 | 0.001 | 0.047 | 0.003 |
| Up (M2) | IPR | IPR041874 | Carbonic anhydrase, CA4/CA15 | 20 | 1 | 3 | 0.001 | 0.056 | 0.003 |
| Up (M2) | GO: MF | GO:0004089 | Carbonate dehydratase activity | 20 | 1 | 4 | 0.001 | 0.057 | 0.003 |
| Up (M2) | KEGG | K08896 | BMX, ETK; cytoplasmic tyrosine-protein kinase BMX [EC:2.7.10.2] | 20 | 1 | 4 | 0.001 | 0.059 | 0.003 |
| Up (M2) | IPR | IPR035875 | BMX, SH2 domain | 20 | 1 | 4 | 0.001 | 0.070 | 0.003 |
| Up (M2) | GO: MF | GO:0005219 | Ryanodine-sensitive calcium-release channel activity | 20 | 1 | 6 | 0.001 | 0.080 | 0.003 |
| Up (M2) | GO: MF | GO:0004910 | Interleukin-1, type II, blocking receptor activity | 20 | 1 | 7 | 0.002 | 0.092 | 0.003 |
| Up (M2) | IPR | IPR003032 | Ryanodine receptor Ryr | 20 | 1 | 6 | 0.002 | 0.097 | 0.003 |
| Up (M2) | IPR | IPR009460 | Ryanodine Receptor TM 4-6 | 20 | 1 | 6 | 0.002 | 0.097 | 0.003 |
| Up (M2) | IPR | IPR013333 | Ryanodine receptor | 20 | 1 | 6 | 0.002 | 0.097 | 0.003 |
| Up (M2) | IPR | IPR035761 | Ryanodine receptor, SPRY domain 1 | 20 | 1 | 6 | 0.002 | 0.097 | 0.003 |
| Up (M2) | IPR | IPR035762 | Ryanodine receptor, SPRY domain 3 | 20 | 1 | 6 | 0.002 | 0.097 | 0.003 |
| Up (M2) | IPR | IPR035764 | Ryanodine receptor, SPRY domain 2 | 20 | 1 | 6 | 0.002 | 0.097 | 0.003 |
| Up (M2) | IPR | IPR004077 | Interleukin-1 receptor type II | 20 | 1 | 7 | 0.002 | 0.111 | 0.003 |
| Up (M2) | GO: BP | GO:1901031 | regulation of response to reactive oxygen species | 20 | 1 | 8 | 0.002 | 0.117 | 0.003 |
| Up (M2) | IPR | IPR006730 | Sestrin | 20 | 1 | 8 | 0.002 | 0.125 | 0.003 |
| Up (M2) | IPR | IPR029032 | AhpD-like | 20 | 1 | 9 | 0.003 | 0.139 | 0.004 |
| Up (M2) | GO: BP | GO:0006874 | Intracellular calcium ion homeostasis | 20 | 1 | 12 | 0.003 | 0.169 | 0.004 |
| Up (M2) | IPR | IPR000699 | RIH domain | 20 | 1 | 12 | 0.003 | 0.181 | 0.004 |
| Up (M2) | IPR | IPR013662 | RyR/IP3R Homology associated domain | 20 | 1 | 12 | 0.003 | 0.181 | 0.004 |
| Up (M2) | IPR | IPR014821 | Inositol 1,4,5-trisphosphate/ryanodine receptor | 20 | 1 | 12 | 0.003 | 0.181 | 0.004 |
| Up (M2) | IPR | IPR035910 | RyR/IP3 receptor binding core, RIH domain superfamily | 20 | 1 | 12 | 0.003 | 0.181 | 0.004 |
| Up (M2) | IPR | IPR040907 | IL-3 receptor alpha chain, N-terminal | 20 | 1 | 15 | 0.004 | 0.222 | 0.005 |
| Up (M2) | IPR | IPR001562 | Zinc finger, Btk motif | 20 | 1 | 25 | 0.007 | 0.360 | 0.007 |
| Up (M2) | IPR | IPR016093 | MIR motif | 20 | 1 | 28 | 0.007 | 0.402 | 0.008 |
| Up (M2) | IPR | IPR036300 | Mir domain superfamily | 20 | 1 | 28 | 0.007 | 0.402 | 0.008 |
| Up (M2) | GO: MF | GO:0004908 | Interleukin-1 receptor activity | 20 | 1 | 42 | 0.009 | 0.492 | 0.009 |
| Up (M2) | GO: MF | GO:0005262 | Calcium channel activity | 20 | 1 | 42 | 0.009 | 0.492 | 0.009 |
| Up (M3) | KEGG | K09455 | MITF; microphthalmia-associated transcription factor | 2 | 1 | 14 | < 0.001 | 0.020 | 0.002 |
| Up (M3) | IPR | IPR031867 | MiT/TFE transcription factors, N-terminal | 2 | 1 | 29 | 0.001 | 0.029 | 0.002 |
| Up (M3) | IPR | IPR021802 | MiT/TFE transcription factors, C-terminal | 2 | 1 | 41 | 0.001 | 0.040 | 0.003 |
| Up (M3) | IPR | IPR011598 | Myc-type, basic helix-loop-helix (bHLH) domain | 2 | 1 | 350 | 0.006 | 0.336 | 0.007 |
| Up (M3) | IPR | IPR036638 | Helix-loop-helix DNA-binding domain superfamily | 2 | 1 | 376 | 0.007 | 0.361 | 0.007 |
| Up (M3) | GO: MF | GO:0046983 | Protein dimerization activity | 2 | 1 | 547 | 0.008 | 0.450 | 0.008 |
| Up (M4) | IPR | IPR021922 | Par3/HAL, N-terminal | 2 | 1 | 21 | < 0.001 | 0.016 | 0.002 |
| Up (M4) | KEGG | K04237 | PARD3; partitioning defective protein 3 | 2 | 1 | 15 | < 0.001 | 0.021 | 0.002 |

These terms were identified using the program FUNC-E, a python package for the functional enrichment analysis of gene sets.

^a^ Module size: The total number of genes in the module.

^b^ Count in Module: The number of genes in the module annotated with the term.

^c^ Count in Background: The total number of genes in the background annotated with the term.

^d^ Fisher’s p-value: The raw p-value from the Fisher’s exact test.

^e^ Bonferroni: The Bonferroni-corrected p-value for multiple testing

^f^ Benjamini: The Benjamini-Hochberg (FDR) corrected p-value for multiple testing.
