# Supplementary figures and images for: Peripheral leukocyte transcriptomic changes in preweaned Holstein heifer calves with varying stages of Bovine Respiratory Disease
Source: PLoS One. 2026 May 14;21(5):e0349348. doi: 10.1371/journal.pone.0349348 (PMC13175367; doi:10.1371/journal.pone.0349348)

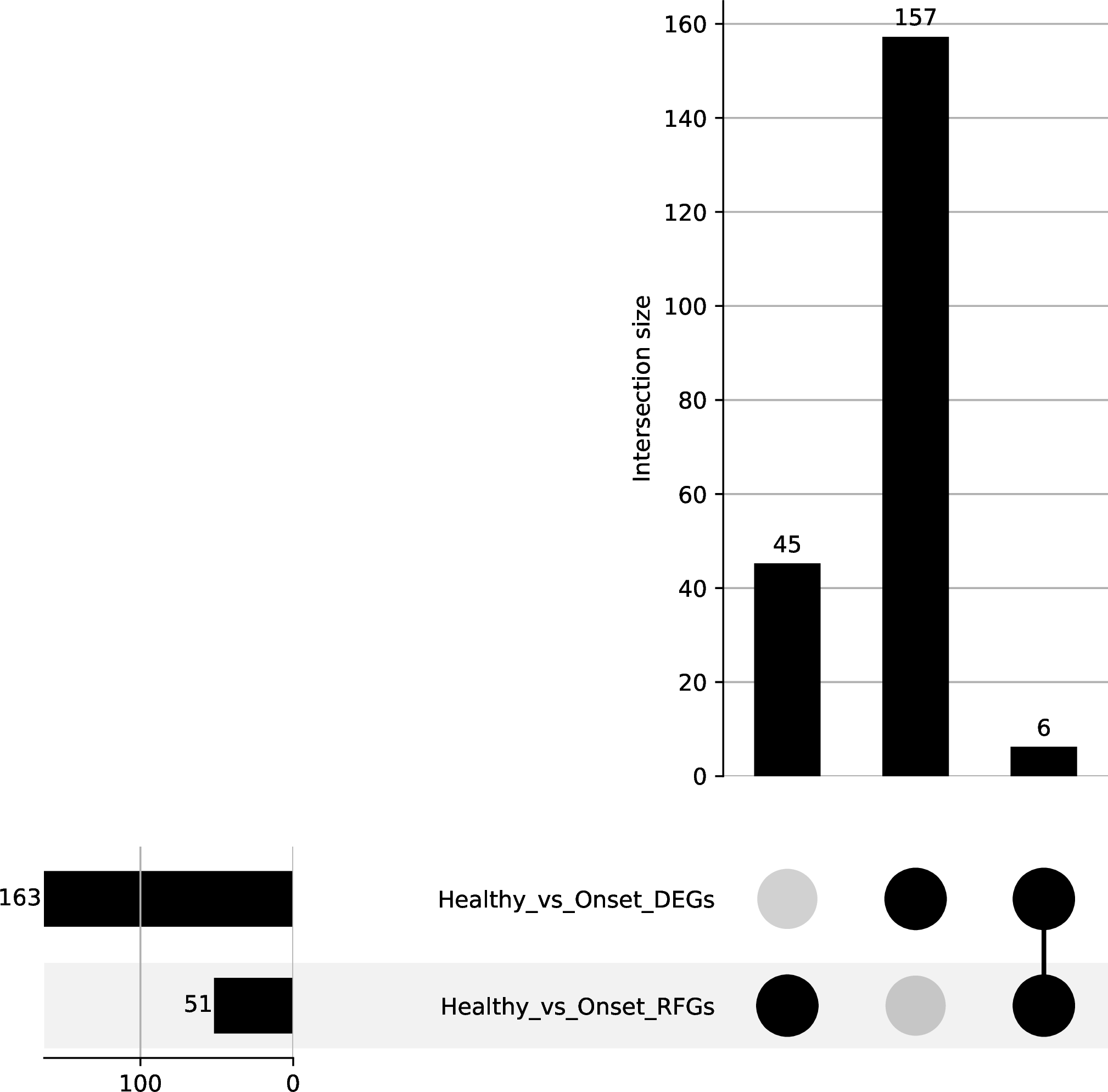

Supplement: S3 Fig — The vertical bars define the intersection size. The horizontal bars show the size of each set. The filled circles in a column set indicates that the set includes an intersection. (TIF) [file pone.0349348.s012.tif]

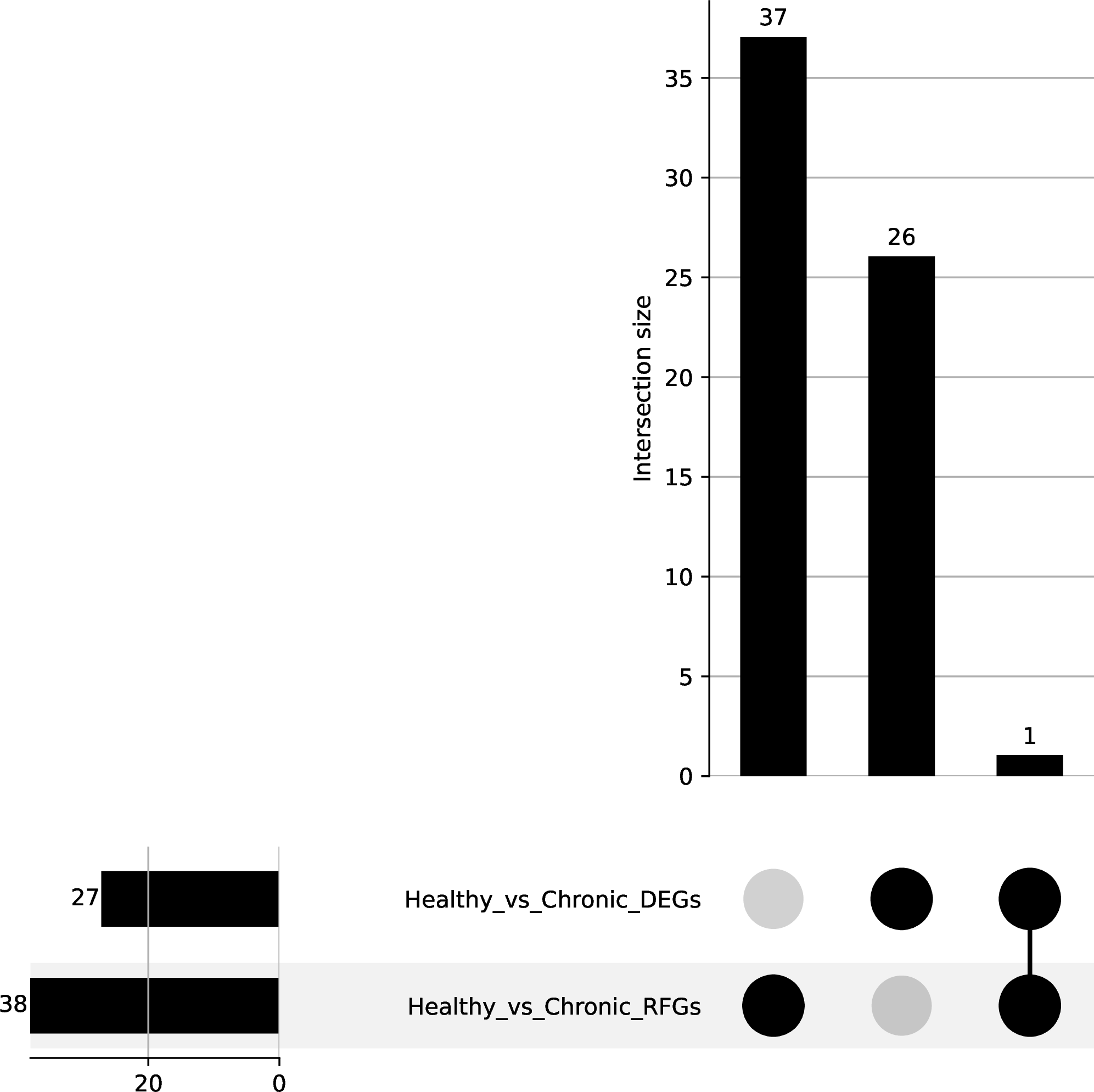

Supplement: S4 Fig — The vertical bars define the intersection size. The horizontal bars show the size of each set. The filled circles in a column set indicates that the set includes an intersection. (TIF) [file pone.0349348.s013.tif]
